# A Sox2–Sox9 signalling axis maintains human breast luminal progenitor and breast cancer stem cells

**DOI:** 10.1038/s41388-018-0656-7

**Published:** 2019-01-08

**Authors:** Giacomo Domenici, Iskander Aurrekoetxea-Rodríguez, Bruno M. Simões, Miriam Rábano, So Young Lee, Julia San Millán, Valentine Comaills, Erik Oliemuller, José A. López-Ruiz, Ignacio Zabalza, Beatrice A. Howard, Robert M. Kypta, Maria dM Vivanco

**Affiliations:** 1 CIC bioGUNE, Technological Park Bizkaia, Derio, 48160 Spain; 2 The Breast Cancer Now Toby Robins Research Centre, The Institute of Cancer Research, London, UK; 3 Radiodiagnostic Service PreteImagen, Bilbao, Spain; 4 Department of Pathology, Galdakao-Usansolo Hospital, Galdakao, Spain; 5 Department of Surgery and Cancer, Imperial College London, London, UK

**Keywords:** Breast cancer, Cancer stem cells

## Abstract

Increased cancer stem cell content during development of resistance to tamoxifen in breast cancer is driven by multiple signals, including Sox2-dependent activation of Wnt signalling. Here, we show that Sox2 increases and estrogen reduces the expression of the transcription factor Sox9. Gain and loss of function assays indicate that Sox9 is implicated in the maintenance of human breast luminal progenitor cells. CRISPR/Cas knockout of Sox9 reduces growth of tamoxifen-resistant breast tumours in vivo. Mechanistically, Sox9 acts downstream of Sox2 to control luminal progenitor cell content and is required for expression of the cancer stem cell marker ALDH1A3 and Wnt signalling activity. Sox9 is elevated in breast cancer patients after endocrine therapy failure. This new regulatory axis highlights the relevance of SOX family transcription factors as potential therapeutic targets in breast cancer.

## Introduction

Breast cancer is a very heterogeneous disease. Analysis of the gene expression profiles of breast carcinomas has revealed the existence of various tumour subtypes with clinical implications [1]. Further studies, including the integrated analysis of copy number and gene expression, have revealed the presence of a novel molecular stratification [2]. These molecular classifications reflect the genetic diversity of breast tumours among patients. However, there is another level of complexity at the tumour level, as each tumour is not a mass of a single type of cell, but a mixture of different cell types, including cells with characteristics of stem/progenitor cells. It appears that both stem cells and luminal progenitors could be targets of transformation, giving rise to different cancer subtypes [3].

The mammary epithelium is composed of two main cellular lineages, luminal and myoepithelial and, in addition, stem and progenitor cells responsible for ductal lobular outgrowth. Various approaches have been used to identify breast cells with characteristics of stem cells, including the expression of specific cell surface markers, such as EMA and CALLA [4] and CD49f and EpCAM (ESA) [5, 6], which identify normal breast stem/progenitor cells. In addition, high CD44 and low CD24 (CD44^+^CD24^−/low^) [7], as well as elevated aldehyde dehydrogenase (ALDH) activity [8] and increased mammosphere-forming capacity [9], have been confirmed as methods that enrich for cells with characteristics of cancer stem cells (CSCs), as assayed by increased tumour initiation potential in transplantation studies. Cells referred to as CSCs or tumour-initiating cells drive tumour initiation and growth and, in addition, CSCs are also more resistant than non-CSCs to current forms of therapy, including radiotherapy [10], chemotherapy [11] and hormone therapy [12].

Studies in our laboratory showed that the increase of CSCs during development of resistance to tamoxifen is driven by enhanced levels of Sox2 [12]. Genetic profiling of Sox2 overexpressing cells [12] revealed increased expression of Sox9 in these cells. Sox9 is a member of the high mobility group (HMG) superfamily of transcription factors that is expressed in progenitor or stem cells in multiple tissues, including the skin, pancreas, intestine and liver [13–16]. In the mammary gland, Sox9 cooperates with the transcription factor Slug to orchestrate the stem cell state [17]. In the mouse, Sox9 has been shown to be a key re-gulator of mammary gland development and stem/progenitor cell maintenance [18] and, in breast cancer patients, high-Sox9 expression has been associated with estrogen receptor (ER)-negative tumours, significantly shorter overall survival and poor survival [19].

Here, we show that Sox9 expression, which is directly induced by Sox2, marks luminal progenitors in normal human breast epithelial cells and in breast CSCs. We further show that Sox9 expression is repressed by estrogen, re-gulates the luminal progenitor population by directly inducing ALDH1A3 expression and that Sox9 knockout using CRISPR restores sensitivity to tamoxifen in vivo. Finally, Sox9 expression is required for Wnt signalling in breast cancer cells. Our observations support a model in which Sox9 is required for the maintenance of luminal progenitors in the human breast and for Wnt signalling in tamoxifen-resistant breast cancer cells.

## Results

### Sox9 expression in normal human breast epithelial cells

**Fig. 1:**
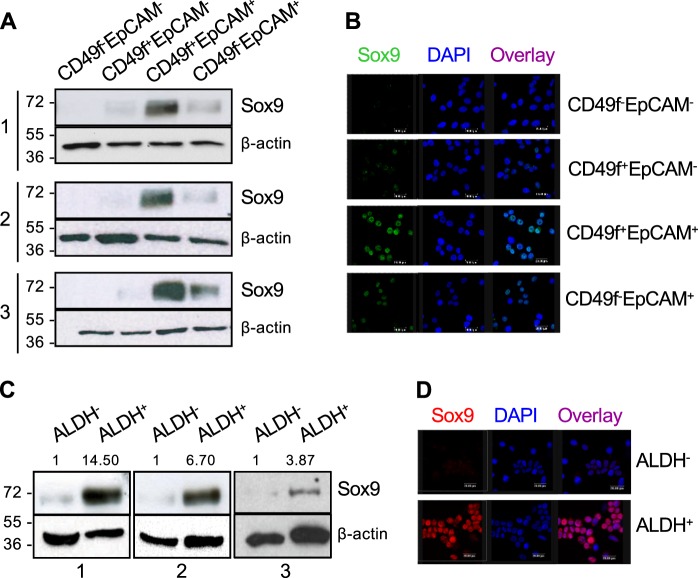
Sox9 marks luminal progenitor cells and ALDEFLUOR+cells in the human breast. **a** Sox9 protein levels in CD49f-EpCAM^−^, CD49f^+^EpCAM^−^, CD49f^+^EpCAM^+^ and CD49f^−^EpCAM^+^ cell populations from three different primary human breast epithelial cell samples were assessed by western blot. **b** Immunofluorescence analysis of Sox9 expression in CD49f^−^EpCAM^−^, CD49f^+^EpCAM^−^, CD49f^+^EpCAM^+^ and CD49f^−^EpCAM^+^ cell populations sorted from primary human breast epithelial cells from one tissue sample, as representative example. **c** Sox9 levels in ALDEFLUOR^−^ (indicated as ALDH^−^) and ALDEFLUOR^+^ (indicated as ALDH^+^) cells sorted from three different human breast epithelial cell samples. **d** A representative example of immunofluorescence analysis of Sox9 expression in ALDEFLUOR^−^ and ALDEFLUOR^+^ cells sorted from human breast epithelial cells

We wished to examine Sox9 expression in different epithelial cell populations in the human mammary gland. To this end, breast epithelial cells were isolated from reduction mammoplasties and FACS sorted according to different phenotypes. First, membrane markers CD49f and EpCAM were used to distinguish between mature luminal cells (CD49f^−^EpCAM^+^), luminal progenitor cells (CD49f^+^EpCAM^+^), myoepithelial/stem cells (CD49f^+^EpCAM^−^) and negative/stromal cells (CD49f^−^EpCAM^−^) [6, 20] (Supplementary Fig. S1a). Western blot (Fig. 1a) and immunofluorescence (Fig. 1b) analyses of FACS sorted cells from five reduction mammoplasties showed that Sox9 is predominantly expressed by the double-positive CD49f^+^EpCAM^+^ luminal progenitor cell subset and, to a lesser extent, by CD49f^−^EpCAM^+^ luminal cells (patient characteristics are shown in Supplementary Table S1). Second, we examined Sox9 levels in cells with increased aldehyde dehydrogenase (ALDH) activity (ALDEFLUOR-positive or ALDH^+^) since these cells have been shown to display stem/progenitor cell properties [8]. Sox9 expression was significantly higher in ALDH^+^ than in ALDH^−^ cells, as observed by western blot (Fig. 1c) and immunofluorescence (Fig. 1d), further indicating preferential expression in stem/progenitor cells.

On the basis of Sox9 upregulation both in luminal progenitors and in ALDH^+^ primary breast epithelial cells, we analysed the degree of overlap of these two cell subpopulations. Indeed, fractionation of ALDH^+^ and ALDH^−^ cell populations from primary human breast epithelial cell samples using CD49f and EpCAM markers showed that the majority of ALDH^+^ cells were found within the luminal progenitor cell population (81.9% ± 5.8% are CD49f^+^EpCAM^+^), with a lower percentage of differentiated luminal cells (CD49f^−^EpCAM^+^ cells, 16.4% ± 5.9%) and an almost negligible number of CD49f^+^EpCAM^−^(0.6% ± 0.2%) or CD49f^−^EpCAM^−^ cells (1.1% ± 0.8%). On the other hand, the ALDH^−^ cells were found mostly in the mixed myoepithelial/stem cell compartment (62.7% ± 8.2%) (Supplementary Fig. S1b). These findings indicate that Sox9 marks ALDH^+^ and CD49f^+^EpCAM^+^ luminal progenitor cells in the human breast.

### Sox9 expression regulates luminal progenitor cell fate

**Fig. 2:**
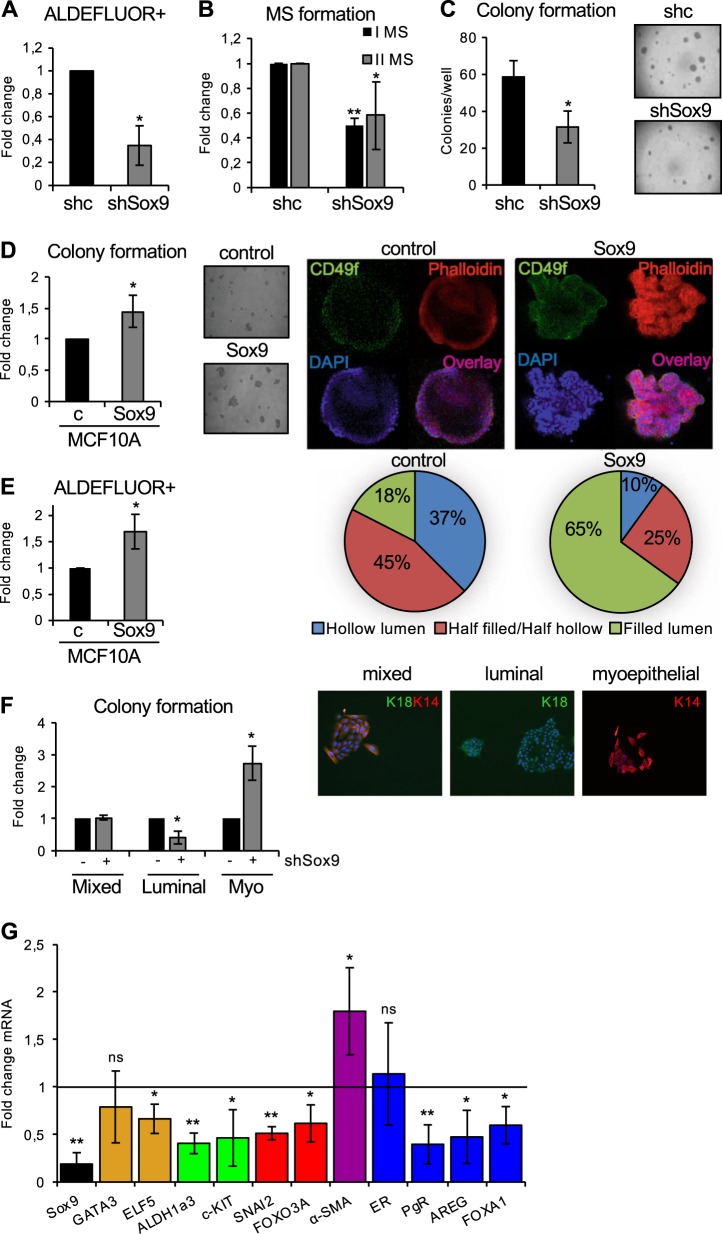
Modulation of Sox9 levels alters human mammary stem cell phenotype. **a** ALDEFLUOR assay in primary breast epithelial cells stably transduced with shcontrol (shc) and shSox9 lentivirus (*n* = 4). **b** Primary (I MS) and secondary (II MS) mammosphere formation in primary human breast epithelial cells transduced with shcontrol (shc) and shSox9 lentivirus (*n* = 5). **c** Colony formation assay on Matrigel of primary epithelial cells stably transduced with shcontrol (shc) and shSox9 lentivirus (*n* = 4). A representative image is shown. **d** Colony formation assay on Matrigel of MCF10A cells stably transduced with plenti6.2-GFP **c** or pLenti6.2-Sox9 (Sox9) (*n* = 4). A phase-contrast (left) and a confocal immunofluorescence (right) images of acini stained for CD49f-APC (green), Phalloidin (red) and DAPI (blue) are shown. The pie graphs show percentage of MCF10A colonies growing as acini in Matrigel displaying different types of lumen (hollow, half-filled/half-hollow and filled). **e** ALDEFLUOR assay in MCF10A cells stably transduced with pLenti6.2V.DEST **(c)** and pLenti6.2-Sox9 (Sox9) (*n* = 5). **f** Luminal (keratin 18, K18^+^), myoepithelial (keratin 14, K14^+^) and mixed (K18^+^K14^+^) colonies formed on collagen-coated wells from human primary breast epithelial cells transfected with shcontrol (−) or shSox9 (+). Results are shown as fold change in number of colonies compared to shcontrol cells (*n* = 3). Representative colony images are shown. **g** Relative transcript levels of the indicated genes in shSox9 primary human breast epithelial cells compared to shcontrol cells (*n* = 4/5). Error bars represent standard deviation (SD). *p < 0.05, **p < 0,001, statistical test: two-tail *t*-test

ALDEFLUOR activity has been shown to be a marker of normal and malignant human mammary stem cells [8] and Sox9 has been implicated in determining the mammary stem cell state [17]. We therefore tested the possible functional role of Sox9 in maintaining the ALDH^+^ cell population in the human mammary gland. Primary human breast epithelial cells from three different donor samples were stably transduced using lentiviral-mediated delivery of control and Sox9 shRNA constructs (Supplementary Fig. S2a). ALDEFLUOR assays showed a significant decrease in the percentage of the ALDH^+^ population in cells with reduced Sox9 levels (Fig. 2a and Supplementary Fig. S2b). Furthermore, stable Sox9 silencing in primary human breast epithelial cells led to reduced primary and secondary mammosphere formation (Fig. 2b), inhibition of cell proliferation in 2D (Supplementary Fig. S2c) and in 3D, with reduced formation of acini in Matrigel, as compared to controls (Fig. 2c). Conversely, overexpression of Sox9 in the breast epithelial cell line MCF10A (Supplementary Fig. S2d) resulted in an increase in colony formation, in the numbers of acini with an irregular shape and filled lumens (Fig. 2d) and in the ALDH^+^ cell population (Fig. 2e and Supplementary Fig. S2e), compared to control-transduced cells, suggesting that Sox9 contributes to the maintenance of the stem/progenitor pool in the human breast epithelium.

In order to determine whether Sox9 expression levels influence the differentiation potential of progenitor cells, the number of multilineage colonies generated by shcontrol and shSox9 breast epithelial cells was tested by culture on collagen. The percentage of cells with bilineage differentiation potential (mixed colonies, K18^+^K14^+^) was unaffected (Fig. 2f). In contrast, silencing of Sox9 clearly reduced the number of luminal colonies (K18^+^) formed, while myoepithelial colonies (K14^+^) increased (Fig. 2f). In addition, colonies were also stained for Muc-1 (as a luminal marker) and p63 (as a basal marker), supporting this finding (Supplementary Fig. S2f). These results support the hypothesis that Sox9 regulates lineage specification by human luminal progenitors. Finally, we analysed expression levels of genes involved in luminal/myoepithelial cell differentiation (GATA3, ELF5 and α-SMA), progenitor cell markers (ALDH1A3 and c-KIT), stemness factors, including FOXO3A and SNAI2/SLUG, and also ER, PR, AREG and FOXA1. Sox9 silencing in primary epithelial cells significantly reduced the expression of progenitor and luminal markers and ER signalling pathway genes, and induced expression of the myoepithelial marker α-SMA (Fig. 2g). Together, these findings indicate that Sox9 is required for the maintenance of the mammary stem/progenitor cell pool in the human breast epithelium and for commitment to the luminal epithelial lineage.

### Sox9 is highly expressed in breast tumours compared to normal tissue

**Fig. 3:**
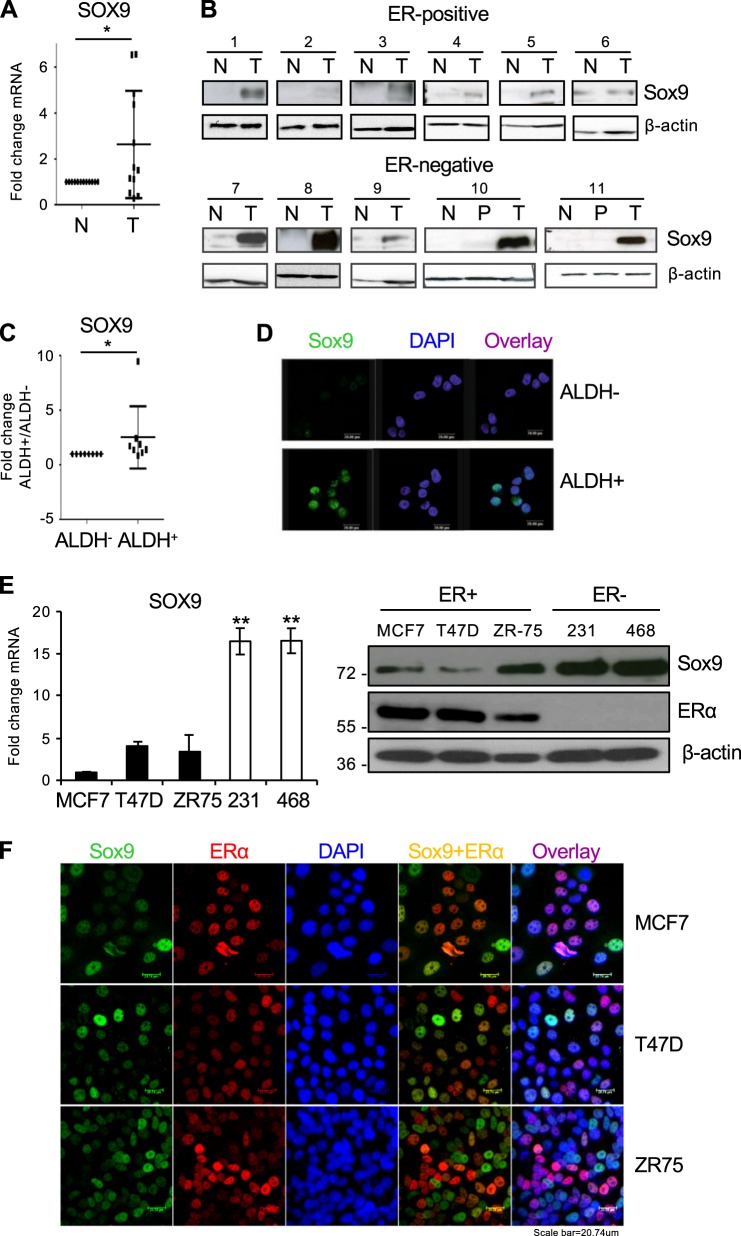
Sox9 is highly expressed in human breast tumours. **a** SOX9 mRNA expression levels in human breast tumour (T) samples compared to their normal (N) counterparts (*n* = 13). **b** Immunoblot of Sox9 and β-actin (loading control) in a set of ER-positive and ER-negative breast tumours (T) compared to the corresponding normal (N) and peritumoral (P) tissue (*n* = 11). **c** Transcript levels of SOX9 in ALDH^−^ and ALDH^+^ cells sorted from 8 different human primary breast tumours. **d** Immunofluorescence analysis of Sox9 expression in ALDH^−^ and ALDH^+^ cells sorted from a primary breast tumour, as representative example. **e** SOX9 mRNA (left) and Sox9 protein (right) levels in ER-positive (MCF7, T47D, and ZR-75–1) and ER-negative (MDA-MB-231 and MDA-MB-468) breast cancer cells, relative to levels in MCF7 cells, set as 1. **f** Immunofluorescence analysis of Sox9 and ER expression in MCF7, T47D and ZR-75-1 breast cancer cells. *p < 0.05, statistical test: Mann–Whitney test **a**; *p < 0.05, **p < 0,001, statistical test: two-tail *t*-test **e**

Sox9 is important for determining the mammary stem cell state both in normal and breast cancer cell lines [17]. We therefore determined whether Sox9 expression levels were altered in breast tumour samples using quantitative real-time PCR and western blotting. Sox9 expression was analysed in a cohort of 30 human primary breast tumours and adjacent normal breast tissue from the same patients. A significant increase in Sox9 expression levels was observed in tumour samples compared to their normal counterparts, both at the mRNA (Fig. 3a) and protein level (Fig. 3b), which also showed the high variability present in primary breast tissues. Additionally, some pairs of normal and tumour samples were probed for the luminal marker Muc-1 in parallel to Sox9, and the results were consistent with Sox9 being more highly expressed in tumour cells than in normal cells (Supplementary Fig. S3a). Similar to our findings in normal breast tissue, ALDH^+^ tumour cells expressed significantly higher levels of SOX9 mRNA (Fig. 3c) and Sox9 protein (Fig. 3d) than ALDH^−^ cells (patient information can be found in Supplementary Table S2). Moreover, analysis of an online database (GSE52327) that compared the expression profiles of ALDH^−^ and ALDH^+^ cell populations isolated from breast cancer samples, also showed higher SOX9 mRNA levels in ALDH^+^ than in ALDH^−^ cells (Supplementary Fig. S3b). These findings suggest that Sox9 could also be expressed in cells with tumour-initiating capacity, since ALDH^+^ cells have been shown to be able of self-renewal and increased tumour generation in xenotransplant models [8, 21].

Despite the relatively small sample number, it appeared that Sox9 expression was strongest in ER-negative tumours (Fig. 3b). To support this observation, we examined SOX9 mRNA levels in publicly available breast tumour datasets. Analysis of a cohort of patients (*n* = 99, GSE2603) confirmed that SOX9 expression is significantly higher in ER-negative than in ER-positive tumours (Supplementary Fig. S3c). Examination of the GOBO database (Gene Expression-Based Outcome for Breast Cancer Online) further confirmed these findings (Supplementary Fig. S3d) and also showed highest SOX9 expression levels in the most aggressive basal/triple-negative breast cancer cell lines (Supplementary Fig. S3e) and reduced recurrence-free survival in patients with basal-like breast cancer (Supplementary Fig. S3f).

Finally, we also examined Sox9 expression in several ER-positive (MCF7, T47D and ZR75-1) and ER-negative (MDA-MB-231 and MDA-MB-468) breast cancer cell lines and found that, as in breast tumour samples, Sox9 is more highly expressed in cell lines lacking ER than in ER-positive cells, both at the mRNA and protein levels (Fig. 3e). Furthermore, immunofluorescence analysis of MCF7, ZR75-1 and T47D cells indicated that those cells with the lowest ER levels expressed the highest Sox9 levels, while cells with strong nuclear ER lacked Sox9 (Fig. 3f). Together, these findings highlight an inverse correlation between Sox9 and ER expression in breast cancer cells and indicate that Sox9 expression is found in both luminal progenitor cells and more aggressive breast tumours.

### ER represses Sox9 expression

**Fig. 4:**
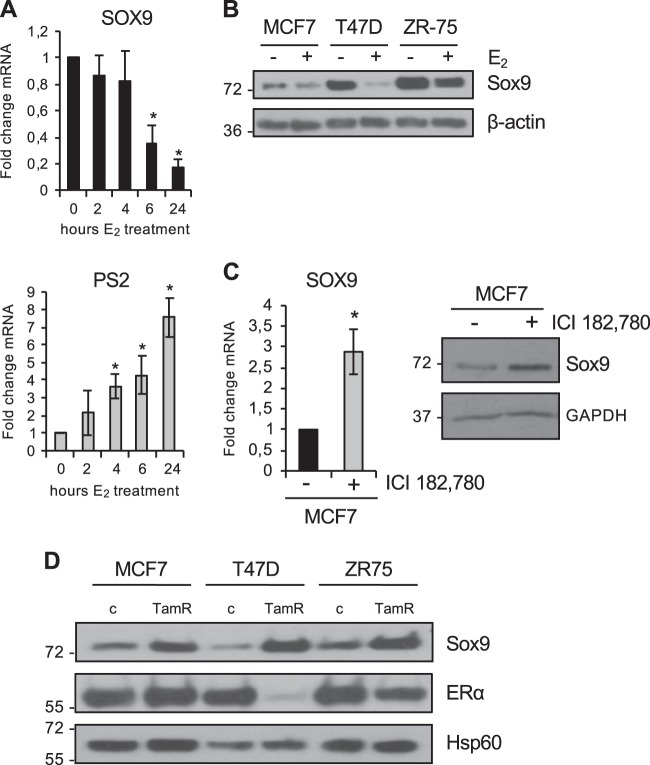
Sox9 expression is repressed by estrogen. **a** Transcript levels of SOX9 and PS2/TFF1 expression in MCF7 cells after 10^−8^ M estrogen (E2) treatment (*n* = 3). **b** Immunoblots of Sox9 in MCF7, T47D and ZR-75-1 cells treated for 2 days with 10^−8^ M estrogen. **c** SOX9 mRNA (left) and Sox9 protein (right) levels after 10^–7^ M ICI 182,780 treatment in MCF7 cells (*n* = 3). **d** Immunoblots for Sox9 in MCF7, T47D, ZR-75–1 and their corresponding tamoxifen-resistant breast cancer cells (parental (**c**) and TamR, respectively). β-actin, GAPDH or Hsp60 have been used as loading controls, as indicated. Error bars represent standard deviation (SD). *p < 0.05, statistical test: two-tail *t*-test, compared to control **a**, **c**

On the basis of the inverse correlation observed between Sox9 and ER expression, we hypothesized that Sox9 expression may be regulated by estrogen in breast cancer cells. Indeed, treatment of MCF7 cells with 10^–8^ M estrogen led to a strong downregulation of SOX9 mRNA in a time-dependent manner, while the expression of PS2, a well-known ER target gene, was increased in parallel (Fig. 4a). This reduction was also observed at the protein level in different ER-positive breast cancer cell lines (Fig. 4b). Furthermore, treatment of MCF7 cells with the ER-antagonist fulvestrant (ICI 182,780), which leads to degradation of ER protein, resulted in a significant recovery of Sox9 expression, both at the mRNA and protein level (Fig. 4c), suggesting that ER is a critical regulator of Sox9 transcription. Furthermore, in silico analysis of GEO datasets confirmed that silencing of ER in MCF7 cells results in enhanced SOX9 mRNA expression (Supplementary Fig. S4a).

Our observations suggest that Sox9 regulation by estrogen may be relevant for the self-renewal or differentiation commitment of stem/progenitor cells. Development of resistance to hormone treatment in ER-positive breast cancer is a major clinical problem in cancer management. We have previously shown that estrogen reduces the progenitor cell pool [22] and that CSCs are implicated in the development of resistance to tamoxifen, a process that compromises ER transcriptional activity [12]. Based on this, it could be hypothesized that Sox9 levels are increased in cells resistant to tamoxifen. Indeed, analysis of three models of tamoxifen resistance developed in our laboratory, based on parental MCF7, T47D and ZR75-1 cells [12], showed that SOX9 mRNA (Supplementary Fig. S4b) and Sox9 protein (Fig. 4d) levels were clearly upregulated in tamoxifen-resistant cells. Together, these findings indicate that ER inhibits Sox9 expression.

These findings raise the possibility that ER directly represses Sox9. ER target genes are regulated by binding of ER to response elements (EREs) [23]. A genome-wide screen for high-affinity binding sites identified a potential ERE near the transcription start site (−2650) of SOX9 conserved in human and mouse [24]. However, chromatin immunoprecipitation (ChIP) assay failed to detect any significant binding of ER to this site of the human SOX9 promoter (Supplementary Fig. S4c), while there was very strong binding to the PS2 promoter, used as a positive control, suggesting that Sox9 regulation by estrogen is not direct. Similarly, stable transduction of ER into ER-negative MDA-MB-231 cells failed to reduce Sox9 expression (Supplementary Fig. S4d), suggesting that additional factor(s) may be required for negative regulation by ER. Moreover, ectopic expression of ER in MDA-MB-231 cells has varied effects on the expression of ER-regulated genes, most likely a result of the absence of co-factors, such as FOXA1 and GATA3, which have been shown to be required to restore estrogen‐responsive growth to MDA-MB-231 cells [25].

### Sox9 silencing impairs stem cell self-renewal

**Fig. 5:**
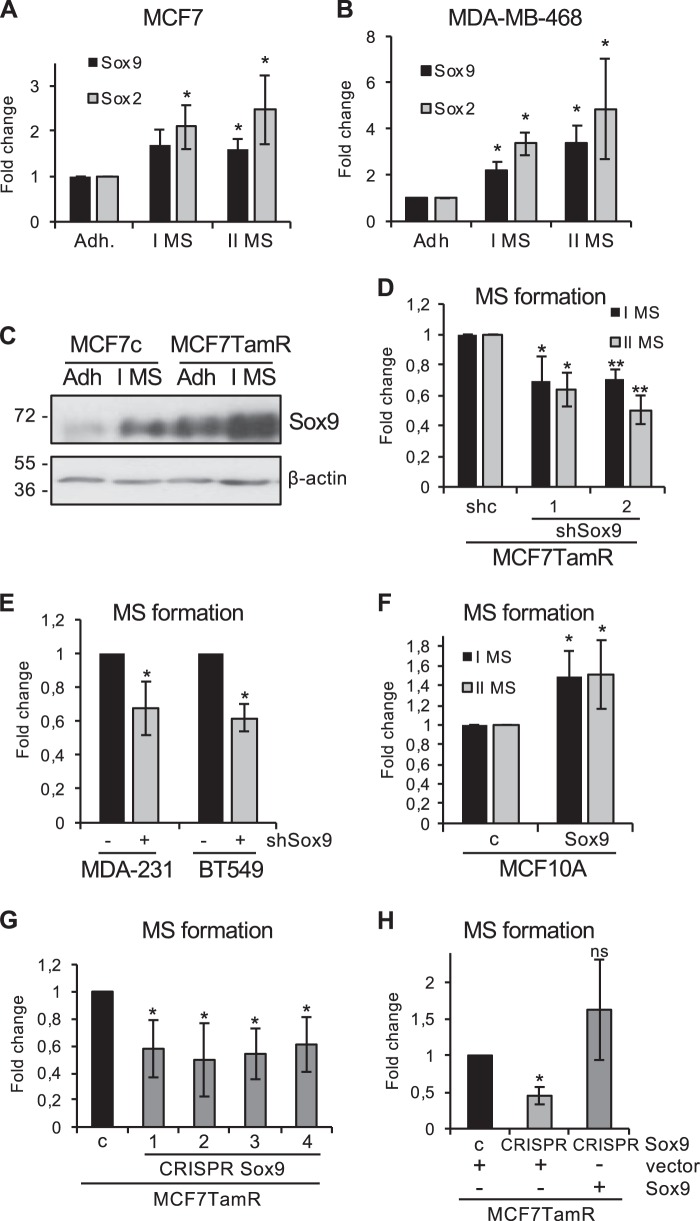
Sox9 regulates breast cancer stem cell renewal. Transcript levels of SOX9 and SOX2 in MCF7 (**a**) and MDA-MB-468 **(b**) breast cancer cells cultured in adherent (Adh) or suspension conditions, as primary (I MS) and secondary (II MS) mammospheres (*n* = 3). **c** Immunoblot of Sox9 and β-actin (loading control) in MCF7 and MCF7TamR cells cultured in adherent (Adh) or suspension (I MS) conditions. **d** Primary (I MS) and secondary (II MS) mammosphere formation in MCF7TamR cells stably transduced with shcontrol (shc) and 2 different shSox9 sequences (1 and 2) lentivirus (*n* = 4/5). **e** Mammosphere formation in MDA-MB-231 and BT549 triple-negative breast cancer cells stably transduced with shcontrol (−) and shSox9 (+) lentivirus (*n* = 3). **f** Primary (I MS) and secondary (II MS) mammosphere formation in MCF10A cells stably transduced with control **c** and Sox9 plasmids (I MS: *n* = 4; II MS *n* = 3). **g** Mammosphere formation in sgRNA binding sense strand only as control (**c**) and four different CRISPR/Cas9n clones using a pair of sgRNAs for both DNA strands, resulting in Sox9 deletion, derived from MCF7TamR cells (*n* = 4). **h** Mammosphere formation in control (**c**) and Sox9 knockout (clone 1) MCF7TamR cells by CRISPR/Cas9n editing (CRISPR Sox9), as in **g**. Control and MCF7TamR cells lacking Sox9 were stably transfected with an empty expression vector (vector) or a vector expressing Sox9 (Sox9) and mammosphere formation was quantified, with the control cells set as 1 (*n* = 3). Error bars represent standard deviation (SD), *p*-value *p < 0.05, **p < 0.001 compared to control **e**, statistical test: two tailed *t*-test **a**, **b**, **e**, **f**, **g**, one-way Anova **d**, **h**

Mammosphere formation capacity and serial passage in suspension can be used to assess stem cell self-renewal, one of the hallmarks of stem cells, and the number of spheres formed in suspension has been shown to correlate with stem cell content [9, 12]. As previously shown for Sox2 [12], Sox9 expression was elevated in ER-positive (MCF7, Fig. 5a), ER-negative (MDA-MB-468, Fig. 5b) and tamoxifen-resistant (MCF7TamR, Fig. 5c) cells cultured in suspension, compared to cells growing in adherent conditions, indicating that Sox9 is upregulated in conditions that enrich for cells with characteristics of stem cells. Furthermore, both primary and secondary mammosphere formation efficiency was diminished in MCF7TamR cells by reducing Sox9 expression levels using two different shSox9 sequences (Fig. 5d and Supplementary Fig. S5a) and also in triple-negative breast cancer cells (Fig. 5e and Supplementary Fig. S5b). Conversely, Sox9 overexpression in MCF10A cells was sufficient to increase primary and secondary mammosphere formation in a modest but statistically significant manner (Fig. 5f). Additionally, deletion of endogenous Sox9 using CRISPR/Cas in MCF7TamR cells (Supplementary Fig. S5c) led to a significant reduction in their capacity for mammosphere formation in all clones tested (Fig. 5g). Finally, this reduction in the ability to form mammospheres was fully restored by exogenous expression of Sox9 (Fig. 5h). These findings suggest that Sox9 is implicated in the self-renewal capacity of progenitor cells in different breast cancer cell types, including in tamoxifen-resistant cells.

### Sox9 regulates ALDH activity through ALDH1A3

**Fig. 6:**
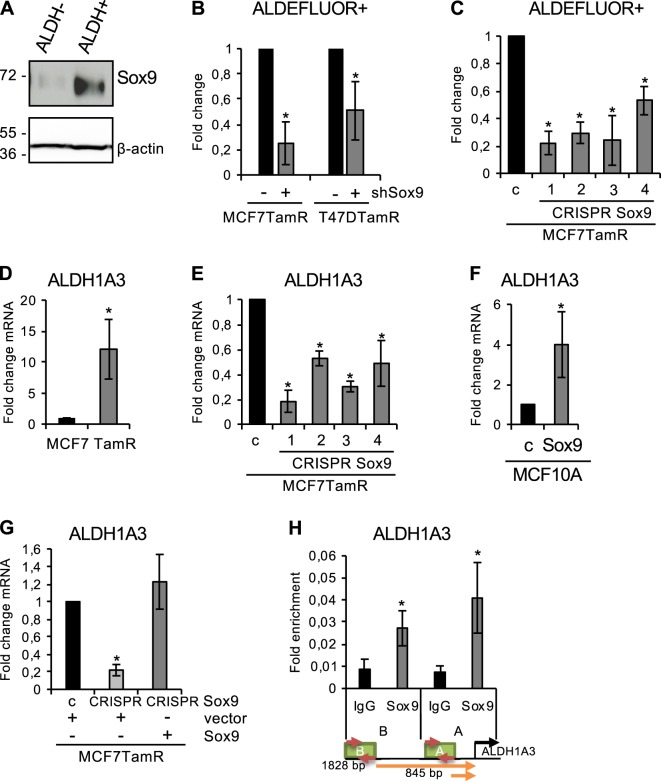
Sox9 expression associates with ALDEFLUOR activity. **a** Immunoblot of Sox9, and β-actin as loading control, in ALDEFLUOR^–^ and ALDEFLUOR^+^ cells sorted from MCF7TamR cells. **b** Fold change of ALDEFLUOR^+^ cells in MCF7TamR and T47DTamR shcontrol (−) and shSox9 (+) cells, (*n* = 4). **c** Fold change of ALDEFLUOR^+^ cells in sgRNA binding sense strand only, as control (**c**) and Sox9 knockout (4 different clones) MCF7TamR cells by CRISPR/Cas9n editing (*n* = 3). **d** ALDH1A3 mRNA expression in parental MCF7 and MCF7TamR (TamR) cells (*n* = 3). **e** ALDH1A3 mRNA expression levels in sgRNA control (**c**) and Sox9 knockout (4 different clones) MCF7TamR cells by CRISPR/Cas9n editing (*n* = 3). **f** ALDH1A3 mRNA expression in MCF10A-GFP (**c**) and MCF10A-Sox9 (Sox9) cells (*n* = 5). **g** ALDH1A3 expression levels in sgRNA control (**c**) and Sox9 knockout MCF7TamR cells by CRISPR/Cas9n editing. The MCF7TamR cells lacking Sox9 were stably transfected with an empty expression vector (vector) or a vector expressing Sox9 (Sox9). **h** Chromatin Immunoprecipitation (ChIP) showing Sox9 binding to human ALDH1A3 promoter in MCF7TamR cells at two positions, A (845 bp) and B (1828 bp) upstream from the transcription start site. Data are shown as fold enrichment compared to IgG binding (*n* = 4). Error bars represent standard deviation (SD). *p < 0.05, compared to control, statistical test: two-tail *t*-test

Next, we wished to investigate further the functional implication of Sox9 in stem/progenitor cell maintenance in tamoxifen-resistant cells. As previously observed in normal and tumour progenitors, Sox9 was predominantly expressed in the ALDH^+^ population isolated from tamoxifen-resistant cells (Fig. 6a). Stable Sox9 silencing using three different shRNA sequences (Supplementary Fig. S6a) significantly reduced ALDH^+^ cell content in MCF7 and T47D tamoxifen-resistant cells (Fig. 6b, Supplementary Fig. S6b). A similar effect was achieved using siRNA oligonucleotides to reduce endogenous Sox9 expression in MCF7TamR cells (Supplementary Fig. S6c). In addition, CRISPR/Cas deletion of Sox9 significantly reduced ALDEFLUOR activity in MCF7TamR cells (Fig. 6c), again confirming the relevance of Sox9 expression for ALDH activity.

ALDH1A3 has been reported to be the most important ALDH isoform responsible for ALDH activity in breast cancer cells, representing a marker of poor prognosis in breast cancer patients [25] and correlating with increased ALDH activity in tamoxifen-resistant cells [12]. Indeed, ALDH1A3 expression was significantly increased in MCF7TamR cells both at the mRNA (Fig. 6d) and protein (Supplementary Fig. S6d) level. Reduction of endogenous Sox9 levels using specific shRNA sequences inhibited ALDH1A3 expression, both in MCF7 and T47D tamoxifen-resistant cells (Supplementary Fig. S6e), as did CRISPR/Cas deletion of Sox9 (Fig. 6e). On the other hand, overexpression of Sox9 in MCF10A cells led to enhanced ALDH1A3 expression (Fig. 6f). To determine whether the reduced ALDH1A3 expression observed by deletion of Sox9 could be rescued, Sox9 was ectopically expressed into Sox9-deficient MCF7TamR cells. Indeed, ectopic Sox9 expression (Supplementary Fig. S6f) was sufficient to restore ALDH1A3 expression levels to those observed in parental cells (Fig. 6g), as well as ALDEFLUOR activity (Supplementary Fig. S6g). Finally, to assess whether Sox9 regulates ALDH1A3 expression directly, ALDH1A3 promoter sequences were analysed using the JASPAR database (http://jaspar.genereg.net/). Chromatin immunoprecipitation showed-specific Sox9 binding to two different regions of the ALDH1A3 promoter (Fig. 6h), similarly to that observed in the TCF4 promoter, which was used as a control (Supplementary Fig. S6h). These findings indicate that Sox9 controls luminal progenitor cell content by regulating ALDH1A3 levels.

### Reduced Sox9 expression enhances tamoxifen sensitivity in vivo

**Fig. 7:**
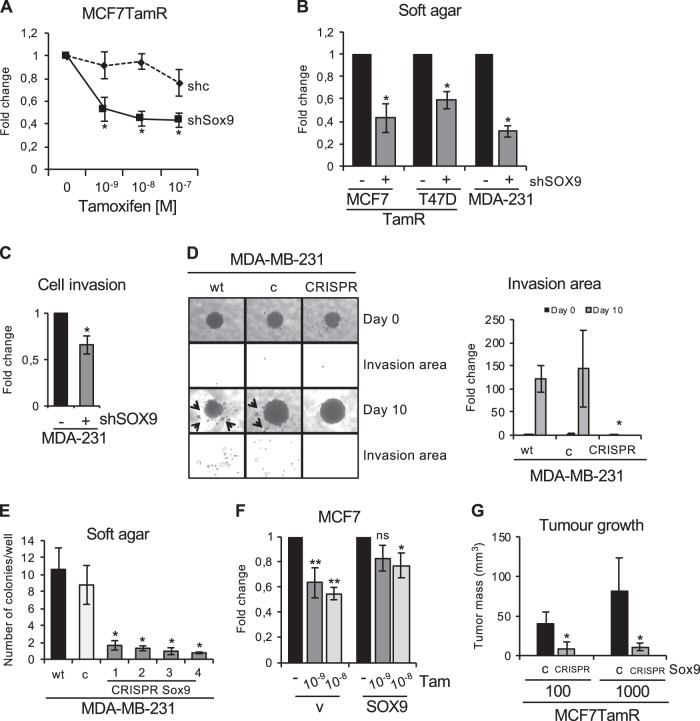
Sox9 expression is implicated in tumorigenicity in vitro and in vivo. **a** Soft agar colony formation assay in MCF7TamR shcontrol (shc) and shSox9 cells with different concentrations of tamoxifen (10^−9^–10^−7^ M), (*n* = 4). **b** Soft agar colony formation assay in MCF7TamR, T47DTamR and MDA-MB-231 shcontrol (−) and shSox9 (+) cells (*n* = 3). **c** Cell invasion assay of MDA-MB-231 shcontrol (−) and shSox9 (+) cells invading through Matrigel in Transwell plates (*n* = 3). **d** Representative images of MDA-MB-231 spheroids grown in Matrigel at the indicated time points from wild type (wt), sgRNA binding sense strand only as control (**c**) and a CRISPR/Cas9n clone using a pair of sgRNAs for both DNA strands, resulting in Sox9 deletion (CRISPR) cells. Below each photograph the analysis of the invaded area by ImageJ is shown and their quantification represented in the graph (*n* = 3). Arrows indicate areas of invasion. **e** Soft agar colony formation assay in wild type (wt), sgRNA control (**c**) and 4 different CRISPR/Cas9n-mediated deletion of Sox9 clones (1–4) in MDA-MB-231 cells (*n* = 3). **f** Soft agar colony formation assay in MCF7 cells stably transduced with an empty vector (v) or a Sox9 expression vector (Sox9) and treated with 10^–9^ M or 10^–8^ M tamoxifen (*n* = 4). **g** Tumour volumes from mammary tumours from each cohort (sgRNA control (**c**) or CRISPR/Cas9n-mediated deletion of Sox9 (CRISPR Sox9) in MCF7TamR cells) collected 18 weeks after injections into mammary fat pad four in NSG female mice in the presence of an exogenous slow oestrogen supplement and a tamoxifen pellet (*n* = 4–6 tumours/group). 100 cells, *p* = 0.0028; 1000 cells, *p* = 0.0423. Mann–Whitney test was used. Statistic test: *t*-test (**a**, **b**, **c**) and one-way Anova (**e**, **f**). Error bars represent standard deviation (SD). *P*, *p*-value: *p < 0.05, **p < 0.001

To determine the effects of Sox9 signalling in breast cancer tumorigenicity, we analysed cell clonogenicity, migration and invasion capacity. Stable Sox9 silencing in tamoxifen-resistant cells significantly enhanced tamoxifen sensitivity (Fig. 7a), suggesting that the number of progenitors, and therefore of hormone-insensitive cells, was reduced. Furthermore, Sox9 silencing reduced the capacity of tamoxifen-resistant cells and triple-negative breast cancer cells to form colonies (Supplementary Fig. S7a), inhibited anchorage-independent growth in soft agar (Fig. 7b) and significantly reduced capacity for invasion (Fig. 7c). In addition, the cell invasion capacity of MDA-MB-231 cells in 3D spheroid cultures was reduced in cells with CRISPR/Cas Sox9 deletion, which only formed tight spheroids that were unable to invade (Fig. 7d, Supplementary Fig. S7b). Furthermore, reduced anchorage-independent growth in soft agar was observed in all deletion clones tested (Fig. 7e). Deletion or silencing of Sox9 in cancer cells led to a clear change in cell morphology, from a characteristic spindle-like morphology to a cobblestone-like monolayer (Supplementary Fig. S7c and S7d). On the other hand, we have previously observed that Sox9 overexpression in non-tumorigenic MCF10A cells was sufficient to induce irregular morphology (Fig. 2d), while ectopic expression of Sox9 in MCF7 cells was sufficient to increase their resistance to tamoxifen (Fig. 7f). We recently reported that Sox9 silencing in MDA-MB-231 cells reduces tumour formation capacity in vivo [17, 26]. To test the effects of Sox9 on tamoxifen sensitivity in vivo, MCF7TamR cells deleted for Sox9 using CRISPR/Cas were orthotopically transplanted into the mammary fat pads of mice. In vivo limiting dilution assay (10^2^, 10^3^, 4 × 10^4^, 10^6^ cells) showed that in contrast to control tamoxifen-resistant cells, Sox9 null cells implanted at low cell density were unable to form substantial tumours in the presence of tamoxifen (Fig. 7g). ELDA (extreme limiting dilution analysis) assay demonstrated that lack of Sox9 significantly reduced the frequency of tumour-initiating cells by 4.76-fold (*p* = 0.00427) in tamoxifen-resistant cells (Supplementary Fig. S7e). Together, the data suggest that Sox9 enhances tumour stemness, leading to increased tamoxifen resistance in vivo.

### Sox9 activates Wnt signalling

**Fig. 8:**
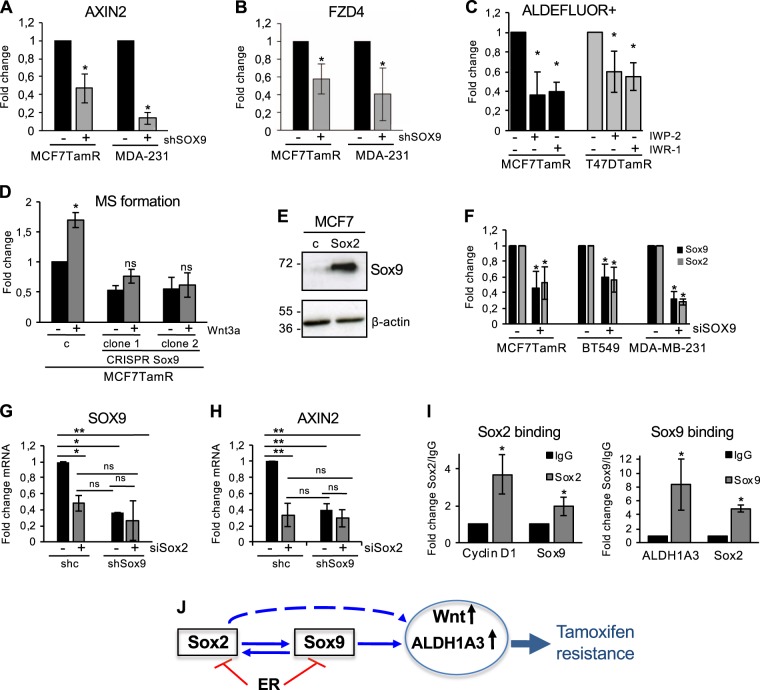
A Sox2–Sox9 axis regulates Wnt activity in breast cancer cells. AXIN2 **a** and Fzd4 **b** mRNA expression levels in MCF7TamR and MDA-MB-231 cells stably transduced with shcontrol (−) and shSox9 (+) lentiviral vectors (*n* = 3/4). **c** Relative change in the proportion of ALDEFLUOR^+^ cells in MCF7TamR and T47DTamR cells treated during 48 h with IWP-2 or IWR-1 Wnt inhibitors (*n* = 3). **d** Mammosphere formation in sgRNA binding sense strand only as control (**c**) and two different CRISPR/Cas9n clones with Sox9 deletion, derived from MCF7TamR cells in the absence (carrier containing CHAPS) or presence of Wnt3a (*n* = 4). **e** Sox9 protein expression levels in control (**c**) and Sox2 overexpressing (Sox2) MCF7 cells, with β-actin as control. **f** Sox2 and Sox9 mRNA expression levels in MCF7TamR, BT549 and MDA-MB-231 cells transiently transfected with sicontrol (−) or siSox9 (+) sequences (*n* = 3). **g** SOX9 and **h** AXIN2 mRNA expression levels in MDA-MB-231 shcontrol (shc) and shSox9 cells transiently transfected with sicontrol (−) and siSox2 (+) sequences (*n* = 3). **i** Chromatin Immunoprecipitation (ChIP) showing Sox2 binding to the human cyclin D1 and SOX9 promoters (left) and Sox9 binding to the human ALDH1A3 and SOX2 promoters (right). IgG control binding is set as 1 (*n* = 3). Error bars represent standard deviations (SD). *p < 0.05, **p < 0.001 compared to control. Statistic test: two-tail *t*-test (**a**, **b**, **c**, **d**, **f**, **g**) and Anova (**i**). **j** Model shows reciprocal regulation between Sox2 and Sox9 leading to activation of ALDH1A3 in breast cancer cells. Dashed arrow shows regulation of Wnt target genes by Sox2 [12], which may involve Sox9 (this report). ER negatively regulates both Sox2 and Sox9. The Sox2–Sox9 axis contributes to increased tamoxifen resistance

Previously we showed that the development of tamoxifen resistance is driven by Sox2-dependent activation of Wnt signalling in cancer stem/progenitor cells [12]. Given the implication of Sox9 in progenitor cells, we determined the effect of Sox9 silencing on two of the Wnt pathway genes identified by microarray [12]. Intriguingly, Sox9 inhibition (whether transient or stable) resulted in significantly reduced expression of AXIN2, a classical Wnt/β-catenin target gene (Fig. 8a and Supplementary Fig. S8a) and of the Wnt receptor and target gene FZD4 (Fig. 8b), in tamoxifen-resistant cells and in triple-negative breast cancer cells. Furthermore, Wnt/β-catenin transcriptional activity, assayed using a TOP/FOP luciferase reporter, was impaired in cells with reduced Sox9 levels, compared to parental cells (Supplementary Fig. S8b).

To investigate further the relationship between Wnt signalling and Sox9, two classes of small molecules that inhibit Wnt signalling were used, the porcupine inhibitor IWP-2, which inhibits Wnt secretion, and IWR-1, which decreases β-catenin levels by stabilising axin. In both cases, inhibition of Wnt signalling resulted in a significant reduction of the progenitor ALDH^+^ cell population (Fig. 8c) in tamoxifen-resistant cells, mimicking the effect observed with Sox9 silencing. On the other hand, addition of Wnt3a to activate Wnt signalling, resulted in an increase in the capacity of MCF7TamR cells to form mammospheres, as expected. However, this increase was not observed in cells with either reduced endogenous levels of Sox9 (Supplementary Fig. S8c) or a total lack of Sox9 (Fig. 8d). Furthermore, while activation of Wnt signalling by the GSK3β inhibitor CHIR99021 increased mammosphere formation in control cells, it was unable to rescue mammosphere formation in cells deleted for Sox9 (Supplementary Fig. S8d), suggesting Sox9 is also required at a point in the Wnt signaling pathway downstream of β-catenin stabilization.

Our previous microarray analysis identified SOX9 as a target of Sox2 [12] and showed that ectopic Sox2 expression leads to increased Sox9 levels (Fig. 8e). We therefore wished to explore further the relationship between these two Sox family factors. Cells with high endogenous levels of Sox2 and Sox9 (MCF7TamR, BT549 and MDA-MB-231 cells) that were transiently silenced for Sox9, presented a significant inhibition in SOX2 expression (Fig. 8f), although this effect was not observed when using cells stably silenced for Sox9 (Supplementary Fig. S8e), suggesting a potential compensatory mechanism to recover SOX2 expression. Conversely, silencing of Sox2 in MDA-MB-231 cells reduced both SOX9 and AXIN2 expression (Fig. 8g, h). Sox2 silencing did not further reduce expression of the Wnt target gene AXIN2 in Sox9-silenced cells (Fig. 8h), suggesting Sox9 may required for Sox2 to activate Wnt signalling in these cells. In addition, in order to explore whether this regulation involved direct DNA binding, chromatin immunoprecipitation was performed. Specific Sox2 binding was observed to consensus DNA binding sequences identified in the SOX9 promoter (http://jaspar.genereg.net/*)* (Fig. 8i). Binding was modest but significant and similar to the observed binding of Sox2 to DNA target sequences identified in the cyclin D1 promoter [27]. On the other hand, specific Sox9 binding was also detected to consensus DNA sequences on the SOX2 promoter (http://jaspar.genereg.net/) (Fig. 8i), confirming that Sox2 and Sox9 can regulate one another at the transcriptional level. Immunofluorescence analysis showed that Sox2 and Sox9 are coexpressed in some cells within the population of tamoxifen-resistant cells (Supplementary Fig. S8f).

Together, these findings suggest a signaling network (Fig. 8j) that includes a regulatory loop in which Sox2 and Sox9 regulate one another, leading to activation of Wnt signalling.

## Discussion

In this study, we demonstrate that Sox9 is implicated in the maintenance of luminal progenitor cells in the human breast and regulates the ALDH^+^ luminal progenitor cell population. Some breast cancers express high levels of Sox9, especially tumours resistant to tamoxifen and ER-negative tumours. This is may be partly due to the observed estrogen-dependent reduction of Sox9 expression in breast cancer cells. Deletion of Sox9 renders tamoxifen-resistant cells unable to form tumours under tamoxifen pressure in vivo. Direct binding of Sox9 to the ALDH1A3 promoter increases the ALDH^+^ cell population. Finally, we report that Sox2 activation of Wnt signalling requires Sox9. These findings reveal a crucial role for Sox9 in maintaining luminal progenitors in the human breast and CSCs in breast cancer.

Sox9 has been shown to be relevant in the regulation and maintenance of stem/progenitor cells in the mouse mammary gland [17]. However, little is known regarding the mechanism of action of Sox9 in stem/progenitor cells in the human breast, and our studies shed light on this issue. Our findings show that CD49f^+^EpCAM^+^ cells are ALDH^+^ and that Sox9 marks both cell populations in human primary breast epithelial cells. ALDEFLUOR-positive cells represent luminal progenitor cells in the human breast [8] and stem cells in the hematopoietic system [28]. CD49f^+^EpCAM^+^ cells have also been identified as luminal progenitor cells, owing to their capacity to undergo differentiation into milk-producing cells upon a lactogenic stimulus [6] and to form budding structures at clonal density [20].

Sox9 and Slug have been shown to cooperate to specify the stem cell state [17] in mice. Immunofluorescence analysis of Slug and Sox9 showed double-positive cells in a small percentage (14.11% ± 2.62%) of luminal progenitor cells (Supplementary Fig. S1c). Here we show that silencing endogenous Sox9 expression in primary human epithelial cell populations was sufficient to reduce luminal progenitor activity. This suggests that Sox9 is important to regulate cell plasticity in the human mammary gland. Indeed, a conditional Sox9 knockout mouse model shows impaired mammary gland formation as well as reduced numbers of luminal mammary progenitor cells [18]. On the other hand, overexpression of Sox9 in vivo increases mammary ductal branching [29]. Together, these findings reinforce the relevance of Sox9 in the human breast for luminal progenitor cell maintenance and differentiation. In addition, Sox9 is also implicated in the maintenance of stem and progenitor cells in neural stem cells [30], pancreatic progenitor cells [31], retinal multipotent mouse progenitor cells [32], lung epithelial progenitors [33], kidney epithelial regeneration [34] and during prostate development [35], suggesting a developmentally conserved role in stem/progenitor cell regulation.

In cancer, however, the role of Sox9 is more diverse. Various studies have found that Sox9 represents a negative prognostic factor in different types of cancer, including glioma [36] and lung [37]. In contrast, Sox9 has been reported to be a tumour suppressor in cervical cancer [38]. Its role in other cancers remains controversial, for example, in melanoma, Sox9 has been reported to be protective [39] or represent a negative prognostic factor [40]. Varied prognostic associations have also been reported in prostate [41, 42], colorectal cancer [43, 44] and bladder cancer [45, 46]. These mixed observations may reflect a context-dependent regulatory role for Sox9 in some tissues.

Our analysis of breast tumour samples reveals elevated levels of Sox9, compared to normal tissue, particularly in patients with ER-negative and in tamoxifen-resistant tumours, as previously described [19, 47]. Analysis of several public datasets confirmed that Sox9 expression is significantly increased in ER-negative and basal-like tumours, in agreement with previous work [19]. Consistent with this, in ER-positive tumour cells, Sox9 does not co-localise with ER. Similar results were found in the normal breast, in which luminal progenitor cells, which express very low levels of ER [6], are enriched in Sox9 expression. This may be partly explained by the observation that estrogen reduces Sox9 expression in ER-positive cells, resulting in high Sox9 levels in ER-negative tumour cells and in tamoxifen-resistant cells, likely due to their reduced ER activity [12]. The hormone-dependent inhibition of Sox9 is in agreement with our previous reports on the effects of estrogen contributing to a more differentiated phenotype by reducing the stem cell pool in the human breast [22, 23] and the association between high CSC content and poorly differentiated tumours [48, 49].

Previous in vivo xenograft studies [17, 26] have shown that Sox9 silencing in MDA-MB-231 breast cancer cells reduces tumour formation. Recently, Sox9 expression was reported to be increased in breast tumour samples after the development of resistance and overexpression of Sox9 in MCF7 cells was shown to confer resistance to tamoxifen in in vitro cell proliferation assays [47]. Our orthotopic tumour transplantations further demonstrate that Sox9 expression is required for tamoxifen resistance in vivo, which may explain the increase in Sox9 levels observed in clinical samples from patients that have developed resistance to tamoxifen.

Enhanced ALDH activity is known to represent stem/progenitor cells in the mammary gland [8]. ALDH1A3 is an important contributor to Aldefluor activity in breast cancer and its expression is predictive of metastasis [25]. Similarly, ALDH1A3 expression is a determinant in malignant pleural mesothelioma cell resistance to chemotherapy [50] and in liver cancer [51], suggesting that it could be a new therapeutic target. We found that Sox9 induces ALDH1A3 expression directly, while its silencing reduces ALDH1A3 mRNA levels, resulting, in both cases, in altered Aldefluor activity. In addition, Sox9 levels control mammosphere formation, suggesting that Sox9 is important for progenitor self-renewal. Consistent with this, the transcriptional profile of human normal mammary stem cells with the capacity to retain the dye PKH26, shows increased SOX9 expression levels [48]. Together, these findings argue for the relevance of Sox9 in maintaining the luminal progenitor cell population in the breast and suggest a cell-of-origin for transformation within this cellular compartment. However, it is important to emphasise that cell plasticity is higher than originally anticipated and cells may be able to alter their differentiation status in response to various intra- and extracellular signals [52].

We previously reported that Sox2 is a marker of breast stem cells [22] and that its expression is reduced by estrogen [12]. The observed increase in SOX9 upon ectopic Sox2 expression and the reduction in SOX2 expression upon silencing of Sox9, together with their reciprocal binding of Sox2 and Sox9 to their respective promoters, suggest a positive regulatory feedback loop between these transcription factors. Both Sox2 and Sox9 silencing reduce Wnt target gene expression and Sox2 silencing reduces expression of SOX9 and AXIN2. However, further inhibition of AXIN2 expression was not observed in the absence of Sox9, suggesting that Sox2 acts upstream of Sox9. Sox2 and Sox9 do not form heterodimers [53], so these effects are unlikely to be mediated by a direct Sox2–Sox9 interaction.

Various pathways have been reported to regulate normal breast and cancer stem cells. Among them, the Wnt signalling pathway appears to be particularly relevant in the breast. Wnt signals are implicated in normal breast development [54] and in maintaining stem/progenitor cells in the human breast [55]. In breast cancer, Wnt signalling is associated with invasion, metastasis and poor survival [56–59]. Notably, we have previously shown that Sox2 activates Wnt signalling in hormone-resistant cells [12] and now find that Sox9, itself a Wnt-responsive gene [15, 60], also regulates Wnt signalling, in agreement with the observed association between Sox9 and the expression of Wnt/β-catenin components LRP6 and Tcf4 in breast cancer [29]. Similarly, it has been shown that Sox9 positively regulates multiple genes required for Wnt signalling in prostate cancer [61]. Furthermore, neither Wnt3a nor CHIR99021 treatment rescued mammosphere formation in cells with reduced levels of Sox9. Together, these observations reveal a positive-feedback loop that implicates Sox2 and Sox9 in the activation of Wnt signalling.

In conclusion, these findings identify a Sox2–Sox9 network as crucial for stem/progenitor cell maintenance in the human mammary gland and warrant further research into the potential of Sox family transcription factors as therapeutic targets in certain types of breast cancer.

## Materials and methods

### Cell culture

MCF7, T47D, ZR-75-1, MCF10A, MDA-MB-231, BT549 and MDA-MB-468 were obtained from the American Type Culture Collection (ATCC). The MCF7, T47D and ZR-75-1 tamoxifen-resistant cell lines were previously developed in the laboratory [12]. MCF10A were cultured as previously reported [62], all the other cell lines were cultured in DMEM:F-12 medium with GlutaMAX (Gibco) supplemented with 8% fetal bovine serum (FBS) and 1% penicillin/streptomycin at 37 °C in 5% CO_2_. For hormone treatments with 17-β-estradiol (Sigma) or Fulvestrant (ICI 182,780), cells were hormone depleted for 72 h in phenol-red free DMEM/F-12 medium supplemented with 8% charcoal-stripped FBS. All cell lines were routinely checked for mycoplasma contamination. Mammosphere cultures were maintained as previously described [12]. When required, Wnt inhibitors were used at 2 µM (IWP-2 and IWR-1) or 1 µM (CHIR99021) final concentration and DMSO was used as vehicle diluted 1/1000 in cell culture medium as control.

### Primary human breast epithelial cells

Normal breast tissue was obtained from women (*n* = 5) undergoing reduction mammoplasty with no previous history of breast cancer (Supplementary table S1) and cells cultured as mammospheres, as previously described [63]. Tumour samples were obtained from core biopsies or from women undergoing therapeutic surgery (*n* = 32) and included paired normal and tumour tissue from the same patient. All samples were reviewed by a consultant breast pathologist. Patients provided written informed consent, and the procedures were approved by the local Hospital Research Ethics Committee and by the ‘Ethics Committee of Clinical Investigation of Euskadi’. The breast tissue was immediately processed as previously described [4, 22].

Stable shRNA clones from primary human breast epithelial cells were cultured with WIT-P-NC^TM^ Medium (Cellaria) supplemented with 1% penicillin/streptomycin. All the experiments using primary breast cells were performed until passage 4 in culture, to avoid signs of reduced cell growth and senescence.

### Growth assays

For differentiation assays, human breast epithelial cells (1000 cells/well) were seeded on glass coverslips previously coated with collagen and cultured with WIT-P-NC^TM^ Medium (Cellaria) supplemented with 1% penicillin/streptomicin. Culture medium was replaced every 3 days in all assays. Formed colonies were fixed in paraformaldehyde 4% for immunofluorescence analysis.

For acinar formation in Matrigel (BD Biosciences), human breast epithelial cells or MCF10A cells, 1000 cells/well in 96-well plates, were seeded on top of a layer of Matrigel. Colonies were counted under the light microscope or stained for immunofluorescence analysis.

For clonogenic assays, breast cancer cells were seeded at 500 cells/well in a 6-well plate. Colonies were fixed and stained with a 0.2% crystal violet, 20% methanol solution and counted.

To assess anchorage-independent growth, soft agar colony formation assays were used. Briefly, 10,000 cells/well, in a 6-well plate, were cultured in triplicate in complete medium with 0.35% low-melting agar over a bottom layer with 0.7% regular agar until visible colonies were formed and counted.

### Lentiviral stable expression

A set of three pLKO.1 lentiviral vector shRNAs targeting Sox9 was purchased from Open Biosystem (source ID: TRCN0000020384, TRCN0000020385, TRCN0000020386). An empty vector and a shRNA against a random sequence, shcontrol, were used as negative controls. Lentiviruses were produced by transfection of 293T packaging cells with a 3-plasmid system. Stably transduced cells were selected by culturing with 2 µg/ml puromycin for 2 days and then maintained in medium containing 0.5 µg/ml puromycin. Stable Sox9 downregulation was assessed by western blot. Sox9 overexpression was achieved using a plenti6.2-GFP (or pLenti6.2-V-DEST for Aldefluor assays) and plenti6.2-Sox9 (kindly provided by Vincent J Hearing, NCI). Briefly, for ER overexpression, ERα cDNA was cloned using XhoI and EcoRI restriction sites into plenti6.2-V-DEST vector.

### Fluorescence activated cell sorting (FACS)

For CD49f/EpCAM stainings, FITC-conjugated anti-EpCAM antibody (Stemcell Technologies, 10110) and APC-conjugated anti-CD49f antibody (eBioscience, 17-0495-80) were used. Control samples were stained with isotype-matched control antibodies, the viability dye 7-aminoactinomycin D (7AAD) (BD) was used for dead cell exclusion, and fluorescence minus one (FMO) controls were used to define the gates [64]. Approximately 100,000 primary cells were FACS sorted and then cytospun on poly-lysine coated slides for immunofluorescence analysis. To measure ALDH activity in cells, ALDEFLUOR assay (Stemcell Technologies) was carried out according to manufacturer’s guidelines, and as previously described [22]. Cells were sorted using a FACSAria (Becton Dickinson) flow cytometer and the data were analysed using the FACSDiva software.

### Western blot

Cell extracts were prepared as previously described [65] and analysed using the following primary antibodies: rabbit anti-Sox9 (Millipore, AB5535), rabbit anti-ALDH1A3 (Abgent, RB16818), mouse anti-GAPDH (Sigma, G8795), mouse anti-β-actin (AC-15/A5441), mouse anti-ERα (Novocastra Leica Biosystems, NCL-ER-6F11), Armenian hamster anti-Muc-1 (ThermoFisher Scientific, MA5-11202), mouse anti-β-tubulin (Sigma, T4026) and rabbit anti-Hsp60 (Santa Cruz Biotechnology, sc-13966). For detection an enhanced chemiluminescence detection kit (Bio-rad) was used.

### Immunofluorescence

Approximately 100,000 primary cells were FACS sorted and then cytospun on poly-lysine coated slides. Cell lines were grown directly on slides and processed as previously described [66]. Briefly, cells were fixed with 4% paraformaldehyde (Santa Cruz Biotech.), permeabilised with 0.5% Triton X-100, blocked for 1 h with 3% BSA and stained with the primary antibodies rabbit anti-Sox9 (Millipore, AB5535), goat anti-Sox2 (Santa Cruz Biotech, sc-17320) and mouse anti-ERα (Novocastra Leica Biosystems, NCL-ER-6F11), and then with anti-rabbit Alexa 488 (Thermo Fisher Scientific, A21206), anti-mouse Alexa 594 (Thermo Fisher Scientific, A21203), anti-goat Alexa-488 (Thermo Fisher Scientific, A11055) secondary antibodies. Finally, slides were mounted in Vectashield with DAPI (Vector Laboratories) and visualized on a Leica confocal microscope.

Differentiated colonies from human breast epithelial cells were stained with the following primary antibodies, mouse anti-K14 (IgG3) (Vector, VPC410) and mouse anti-K8/18 (IgG1) (Bio Rad, MCA1864HT) or Armenian hamster anti-Muc-1 (Thermo Fisher Scientific, MA5-11202) and mouse anti-P63 (Ventana, 790–4509) and secondary antibodies, anti-mouse IgG3 Texas-Red (Southern Biotech, 1100–07) and anti-mouse IgG1 FITC (Southern Biotech, 1070–02) or anti-Armenian hamster Cy3 (Jackson Immuno Research, 127-165-160) and anti-mouse Alexa 488 (Thermo Fisher Scientific, A21202).

MCF10A cell acini were stained according to the protocol from Joan Brugge’s lab [67]. Briefly, MCF10A acini growing on Matrigel were fixed with 4% paraformaldehyde for 20 min at 4 °C, followed by permeabilisation with 0.5% Triton X-100, 10 min at 4 °C and blocked with 3% BSA. Acini were incubated overnight with a rat anti-human CD49f APC-conjugated antibody (eBIOscience, 17-4321-41), followed by DAPI (Sigma) and Phalloidin-TRITC staining.

In order to analyse Sox9 and Slug co-localization, cells were fixed with 100% methanol for 15 min at 4 °C, followed by blocking with 3% BSA-0.1% Triton X-100 in PBS and stained overnight with primary Sox9 antibody. Two rounds of 1 h of the secondary antibody anti-rabbit Alexa-594 incubation were performed. Then, cells were blocked with 5% BSA-PBS for 6 h prior to overnight rabbit anti-Slug (Thermo Fisher Scientific, PA1-86737) primary antibody incubation, followed by 20 min incubation with anti-rabbit Alexa488 antibody. Finally, slides were mounted in Vectashield with DAPI (Vector Laboratories) and analysed using a Leica confocal microscope.

### Transient transfections

Transient transfections and luciferase assays were performed as previously described [12]. Briefly, 40,000 cells were seeded in triplicate in 24-well plate and the following day transfected. The reporter plasmids used included 8XTOPflash and 8XFOPflash, SOX-luciferase, with seven copies of the AACAAAG SOX-binding element or the control SAC-luciferase, with seven copies of the CCGCGGT sequence as negative control (both kindly provided by Dr Philippe Jay, IGF, Montpellier) reporter plasmids and pRL β-galactosidase as control for transfection efficiency. Results are shown as TOP/FOP transcriptional activity in shSox9 cells versus shcontrol cells, set as 1.

Small interfering RNA oligonucleotides were transfected using Lipofectamine 2000 or Lipofectamine RNAiMax (Invitrogen) following the guidelines of the manufacturer and as previously described [52]. Silencing was confirmed by western blot or qRT-PCR. The RNAi sequences used are:

sicontrol (commercial siMISSION, Sigma);

siSox9 (1):UGAAGAAGGAGAGCGAGGAGGACAA;

siSox9 (2):UUGUCCUCCUCGCUCUCCUUCUUCA and

siSox2: CCUGUGGUUACCUCUUCCUCCCACU.

### Real-time polymerase chain reaction (qPCR)

RNA was isolated using the Machery-NagelNucleoSpin® RNA, according to instructions of the manufacturer. Real-time PCR was performed on a ViiA 7 or a QuantStudio 6 Flex Real-Time PCR Systems (Applied Biosystems) and as previously described [12]. Primer (Invitrogen) sequences can be found in Supplementary Table S3.

### Invasion assay

Invasion and migration assays were performed in a 24-well BD FalconTM HTS Multiwell Insert System containing an 8 µm pore size PET (PolyEthyleneTerepthalate) membrane, as previously described [12].

### Chromatin immunoprecipitation (ChIP)

ChIP analysis was performed following manufacturer´s instructions (SimpleChIP® Enzymatic Chromatin IP Kit, Magnetic beads, Cell Signaling). Briefly, at least 10^7^ cells were cross-linked with 1% formaldehyde and the reaction quenched by 1 M glycine. Cells were lysed and nucleic acids were digested using Micrococcal nuclease for 20 min at 37 °C. Digestion was followed by sonication to shear chromatin and storage at −80 °C for subsequent chromatin immunoprecipitation. Chromatin was subjected to RNAse and Proteinase K treatment and followed by DNA purification. Chromatin was incubated overnight with control rabbit IgG and Sox9 antibody (AB5535, Millipore), and a positive control against Histone 3 (H3) was used to check the enrichment of the RPL30 gene. Before immunoprecipitation, 2% of the diluted chromatin was removed and stored at −20 °C for subsequent DNA purification and used as “chromatin input”. The next day, protein G-magnetic beads were added to the chromatin—antibody solution. Chromatin was eluted and protein-DNA crosslink reversal was obtained using Proteinase K. Bounded DNA was purified and qRT-PCR was carried out using a ViiA 7 qPCR system (Applied Biosystems) using primers that amplify the predicted Sox9 binding region in the ALDH1A3 promoter as follows: “A” site F: GATTAGCAGCAAAGGTCTCATGT, R: ACACCGCCTTCCATCCCAGA; “B” site: F: GGAGCAGAGTTCTAAGCTCAA, R: GAAATTATGTCACTGCCAGG. Sox9 binding in Sox2 promoter: F: GTAAGAGAGGAGAGCGGAAGAG, R: CGGCTGTCCAACTCGTATTTCT. Sox2 binding in Sox9 promoter: F: CCAGAGTGGAGCGTTTTGTC, R: TGTCTGGGGGAGAGTTTGCTA. Sox2 binding site in Cyclin D1 promoter: F: TGCCGGGCTTTGATCTTT, R: CGGTCGTTGAGGAGGTTGG. ERα binding site on PS2 promoter: F: TGGGCTTCATGAGCTCCTTC, R: TTCATAGTGAGAGATGGCCGG. Putative ERα binding site on Sox9 promoter: F: TGAACATCAGGAGCGGGTT, R: ATTCAGGGGCTCCATTCGCT.

### Sox9 targeting using CRISPR-Cas9n technology

A pair of sgRNA primers targeting the first Sox9 exon were designed and cloned into the nickase plasmid pSpCas9n(BB)−2A-Puro (PX462, Addgene). sgRNA oligo sequences were: sgRNA A, 5′-TTCAGATCGGGCTCGCCCTT-3′ and B, 5′**-**CCCCGTGTGCATCCGCGAGG-3′. Cells were transiently transfected with the resulting Cas9n vector together with 1 (for the control) or the 2 sgRNA sequences against Sox9, using Lipo2000 reagent (Invitrogen^®^), according to manufacturer’s instructions. Two days after transfection, stably transfected cells were selected with 2 μg/ml puromycin and single-cell clones were picked, subcultured and amplified. DNA was extracted using the QuickExtract^TM^ DNA Extraction Solution (Epicentre) for sequencing, according to manufacturer’s instructions. Genotyping PCR was performed using 2 primers flanking the Cas9n target site (F: 5′-CCGTCGGGCTCCGGCTCGGAC-3′, R: 5′-CTCCAGAGCTTGCCCAGCGTC-3′).

### Spheroid formation assay

In order to avoid cell adherence to the plastic surface, round bottom 96-well plates (Sarsted) were coated with 200 μl of poly(2-hydroxyethylmethacrylate) (poly-HEMA [Sigma]) and dried overnight at 56 °C. The next day, 5000 cells were seeded in duplicate in 200 μl of complete medium. Spheroids were allowed to form for 4 days and after careful medium removal, 50 μl of Matrigel growth factor reduced (BD) was added on the top of the spheroids, allowed to solidify and covered with medium, which was replenished every 3–4 days. Spheroid growth and cell invasion was followed daily, and the images were captured using a camera connected to an inverted microscope. The invaded area was quantified using ImageJ software.

### Mammary fat pad xenotransplantation

NSG mice, purchased from Charles River (Harlow, UK), were housed in individually ventilated cages on a 12-h light/dark cycle, and received food and water ad libitum. All work was carried out under UK Home Office projects and personal licenses following receipt of local ethical approval from the Institute of Cancer Research Ethics Committee and in accordance with local and national guidelines. MCF7TamR cells were suspended in 100 μl of PBS/Matrigel (1:1) and injected into mammary gland 4 of 10–12-week-old female mice, which simultaneously received a 60-day slow release pellet containing 0.72 mg of 17β-estradiol with 5 mg tamoxifen pellet (Innovative Research of America). Cells were injected at varying numbers ranging from 100 to 1 million cells/mouse). Pellets were replaced once. Animals were observed once a week. Tumour volumes were calculated with the formula: (Average (Rmax, Rmin)^3^) × 0.5236, where Rmax and Rmin are the maximum and minimum tumour radii, respectively. The tumour-initiating frequency was used for calculation of frequency of cancer stem cells using the extreme limiting dilution analysis (ELDA) software web interface (http://bioinf.wehi.edu.au/software/elda).

### Statistical analysis

Data from at least three-independent experiments are expressed as means ± standard deviation, SD. Each data point of real-time PCR, mammosphere formation, luciferase activity and proliferation assays were run at least in triplicates and independent experiments were performed at least three times. Student’s *t*-test or Anova were used to determine statistically significant differences and *p* < 0.05 was considered to be statistically significant unless otherwise specified.

## Supplementary information

Sup Info text

Sup Fig 1

Sup Fig 2

Sup Fig 3

Sup Fig 4

Sup Fig 5

Sup Fig 6

Sup fig 7

Sup Fig 8
