# Supplementary material for: A Sox2–Sox9 signalling axis maintains human breast luminal progenitor and breast cancer stem cells
Source: Oncogene. 2019 Jan 8;38(17):3151–69. doi: 10.1038/s41388-018-0656-7 (PMC6756022; doi:10.1038/s41388-018-0656-7)
Supplement: Supplementary file 1 — Sup Info text [file 41388_2018_656_MOESM1_ESM.docx]

**A Sox2–Sox9 signalling axis maintains human breast luminal progenitor and cancer stem cells**

Giacomo Domenici^1^, Iskander Aurrekoetxea-Rodríguez^1^, Bruno M Simões^1^, Miriam Rábano^1^, So Young Lee^1^, Julia San Millán^1^, Valentine Comaills^1^, Erik Oliemuller^2^ José A López-Ruiz^3^, Ignacio Zabalza^4^, Beatrice A Howard^2^, Robert M Kypta^1,5^, Maria dM Vivanco^1*^

**Supplementary Information**

**Contents:**

**Page**

Supplementary Table S1 2

Supplementary Table S2 3

Supplementary Table S3 4

Supplementary Figure Legends S1 to S8 5

**Supplementary Table S1**

Clinical data of patients participating in the study. Samples were obtained from women undergoing reduction mammoplasties with no previous history of breast cancer. nk, not known.

| Sample | Age | Day of menstrual cycle | Number of children | Number of Breast-fed children | Contraceptive pill intake |
| --- | --- | --- | --- | --- | --- |
| 1 | 24 | 21 | 0 | 0 | No |
| 2 | 29 | 24 | 3 | 0 | No |
| 3 | 42 | 22 | 1 | 1 | No |
| 4 | 15 | nk | 0 | 0 | No |
| 5 | 19 | nk | 0 | 0 | No |

**Supplementary Table S2**

**a** Histopathological data of primary tumour samples analysed for Sox9 protein expression by immunoblot in Fig. 3b. ER: estrogen receptor, PR: progesterone receptor, HER-2: human epidermal growth factor receptor 2.

**b** Histopathological data of primary tumour samples analysed for Sox9 mRNA expression in ALDEFLUOR^-^ and ALDEFLUOR^+^ cells in Fig. 3c. ER: estrogen receptor, PR: progesterone receptor, HER-2: human epidermal growth factor receptor 2. nk: not known.

**Supplementary Table S3**

Sequence of oligonucleotides used for qPCR analysis during the study.

**Supplementary figure legends**

**Supplementary Figure S1**

**a** Representative FACS plot of CD49f^-^EpCAM^-^, CD49f^-^EpCAM^+^, CD49f^+^EpCAM^+^ and CD49f^+^EpCAM^-^ cell populations found in primary human breast epithelial cells (left) and percentages of each population (right). **b** Representative FACS plots show that ALDEFLUOR+ cells in the human breast are mostly CD49f^+^EpCAM^+^ luminal progenitor cells. Error bars represent standard deviation (SD). SSC-A: side scatter. **c** Immunofluorescence analysis of Sox9 and Slug expression in CD49f^-^EpCAM^-^, CD49f^+^EpCAM^-^, CD49f^+^EpCAM^+^ and CD49f^-^EpCAM^+^ cell populations sorted from primary human breast epithelial cells from one tissue sample, as a representative example.

**Supplementary Figure S2**

**a** SOX9 mRNA expression in human primary breast epithelial cells stably transduced with shcontrol (shc) and shSox9 lentivirus (n=5). **b** Representative FACS plots show ALDEFLUOR+ cells in shcontrol (c) and shSox9 human breast epithelial cells obtained from 3 different tissue donors. **c** Cell proliferation assay (crystal violet assay) using human primary breast epithelial cells transduced with a control shRNA (shc) and shSox9 (n=3). **d** Immunoblot of Sox9 in MCF10A cells stably transduced with pLenti-6.2-GFP (c) and pLenti-6.2-Sox9 (Sox9). **e** Representative FACS plots show ALDEFLUOR^+^ cells in control (c) and overexpressing Sox9 MCF10A cells. Error bars represent standard deviation (SD). **p*<0.05, ***p*<0.001, statistical test: two-tail *t*-test (a), one-tail *t*-test (c). **f** Luminal (Muc-1), myoepithelial (p63) and mixed (Muc-1 and p63) colonies formed on collagen-coated wells from shcontrol (-) or shSox9 (+) human primary breast epithelial cells. Results are shown as fold change in number of colonies compared to shcontrol cells (n=1). Representative colony images are shown. Scale bar 50 μm.

**Supplementary Figure S3**

**a** Immunoblot of Sox9 and Muc-1 (luminal marker) (left) and their quantification graphs (right) in a set of ER-positive (4, 5) and ER-negative (10) breast tumours (T) compared to the corresponding normal (N) tissue (n=3).  **b** SOX9 and ALDH1A3 mRNA expression levels in ALDEFLUOR^-^ and ALDEFLUOR^+^ cell populations analysed in a public GEO2R dataset containing 8 different primary breast tumour specimens (GSE52327). **c** SOX9 expression in ER-negative and ER-positive tumours in a public GEO2R dataset (GSE2603). SOX9 mRNA expression in ER-negative and ER-positive tumours (**d**) and in breast cancer cell lines (**e**) in the GOBO database. **f** Kaplan–Meier survival analysis shows reduced recurrence-free survival of basal-like breast cancer patients with tumours that express high levels of SOX9. Patients with high (red) and low (black) expression are indicated. Hazard ratio (HR) and *p* value (log rank p) is depicted for the survival analysis. *P* value of <0.05 was considered to be statistically significant. TN: triple negative tumours, HR: hormone responsive, HER2: human epidermal growth factor receptor 2 overexpressing tumours, RFS: relapse free survival.

**Supplementary Figure S4**

**a** SOX9 and ERα expression in MCF7 cells transiently silenced for ERα in a public database (GSE27743). siC: scramble siRNA, siERα: siRNA against ERα. **b** Transcript levels of SOX9 in MCF7, T47D, ZR-75-1 and their corresponding tamoxifen resistant breast cancer cells (parental (c) and TamR, respectively). **c** Chromatin Immunoprecipitation (ChIP) showing ERα binding to human PS2 and SOX9 promoters in MCF7TamR cells in the presence of 10nM estrogen for 6 hours. Data are shown as fold enrichment compared to IgG binding (n=4). **d** SOX9 mRNA (left) and Sox9 protein (right) levels in ER-negative (MDA-MB-231) breast cancer cells overexpressing ERα. Error bars represent standard deviation (SD). **p*<0.05, compared to control (b, c).

**Supplementary Figure S5**

**a** Representative phase-contrast photographs of mammospheres formed by shcontrol (shc), shSox9 (1 and 2) MCF7TamR cells. **b** Immunoblot of Sox9 levels in shcontrol (shc) and shSox9 stably transduced MDA-MB-231 and BT549 cells. β-actin and GAPDH have been used as loading controls. **c** Western blot of Sox9 levels in sgRNA control (c) and 4 different CRISPR/Cas9n KO clones derived from MCF7TamR cells. GAPDH has been used as loading control.

**Supplementary Figure S6**

**a** Immunoblot of Sox9 in shcontrol (shc) and 3 different shSox9 sequences (1-3) MCF7TamR cells and in shcontrol (shc) and shSox9 T47DTamR cells. β-actin, as loading control. Relative ALDEFLUOR+ change in shSox9 (2) and (3) compared to shCNTR cells (shc), set as 1 (n=3). **b** Representative FACS plots and graph of ALDEFLUOR^+^ cells in shcontrol (shc) and shSox9 (1- 3; 3 different sh sequences) MCF7TamR cells (n=4). **c** ALDEFLUOR^+^ cells in MCF7TamR transiently transfected with sicontrol (sic), and two different siSox9 sequences. **d** Representative immunoblot of ALDH1A3 levels in MCF7 control (c) and MCF7TamR (TamR) cells. GAPDH was used as loading control. **e** ALDH1A3 mRNA levels in shcontrol (-) and shSox9 (+) MCF7TamR and T47DTamR cells. (n=4). **f** Immunoblot of Sox9 in sgRNA control (c) and CRISPR/Cas9n KO in MCF7TamR cells (CRISPR Sox9) transiently transfected with an empty plenti6.2-CMV vector or a plenti6.2-Sox9 plasmid. β-actin was used as loading control. **g** ALDEFLUOR activity in sgRNA control (c) or with Sox9 deleted by CRISPR/Cas9n editing (CRISPR Sox9) MCF7TamR cells, transfected with an empty (vector) or Sox9 expression vector (Sox9) (n=4) and FACS plots of ALDEFLUOR activity in MCF7TamR cells stably transfected with shcontrol (shc) or shSox9 lentivirus and then rescued by Sox9 expression. **h** Chromatin Immunoprecipitation (ChIP) of Sox9 binding to human TCF4 promoter in MCF7TamR cells, at the position -372/-212 from the transcription starting site (Wang et al., 2013). Data are shown as enrichment compared to IgG binding (n=3). Error bars represent standard deviation (SD). **p*<0.05. Statistical test: two tail *t-test* (a,c,e,g). shc: scramble shRNA as control, shSox9: shRNAs against Sox9.

**Supplementary Figure S7**

**a** Clonogenicity assay in 2D using MCF7TamR, T47DTamR, MDA-MB-231 and BT549 shcontrol (-) and shSox9 (+) cells (n=3). Representative images of MDA-MB-231 and BT549 shcontrol (shc) and shSox9 crystal-violet stained colonies are shown (right). **b** Representative immunoblot of Sox9 levels in MDA-MB-231 wild type (wt), sgRNA control (c) and Sox9 deleted by CRISPR/Cas9n editing (CRISPR Sox9) cells. β-actin was used as loading control. **c** Phase-contrast photographs of shcontrol (shc) and shSox9 MDA-MB-231 and BT549 cells. **d** Phase-contrast photographs of MDA-MB-231 sgRNA (c) and CRISPR Sox9 cells. **e** Single-cell suspensions of MCF7TamR cells control and CRISPR/Cas deletion of Sox9 injected into the mammary fat pads of mice in limited dilution (100, 1,000, 40,000 or 1 million cells). Measurement of tumour-initiating cell frequency by *in vivo* ELDA. Error bars represent standard deviation (SD). **p*<0.05. Statistical test: two-tail *t*-test.

**Supplementary Figure S8**

**a** SOX9 and AXIN2 mRNA expression in BT549 and MDA-MB-231 cells transiently transfected with sicontrol (sic) and siSox9 sequences (n=3). **b** TOP/FOP transcriptional assay in shcontrol (-) and shSox9 (+) BT549 and MDA-MB-231 cells. **c** Mammosphere formation in shcontrol (shc) and shSox9 MCF7TamR cells in the presence or absence of 100 ng/ml recombinant human Wnt3a. Control cells were treated with the equivalent volume of carrier containing CHAPS (n=5). **d** Mammosphere formation in MCF7TamR (wt) and Sox9 deleted cells (CRISPR) in the absence or presence of the GSK3 inhibitor CHIR99021 at 1μM (n=3). **e** SOX2 and SOX9 expression in shcontrol (-) or shSox9 (+) BT549, MDA-MB-231 or MCF7TamR cells. **f** Representative immunofluorescence of endogenous Sox2 and Sox9 expression in MCF7TamR cells. Error bars represent standard deviation (SD). **p*<0.05. Statistical test: two-tail *t*-test (a, b, c, d).
