# Supplementary figures and images for: A Sox2–Sox9 signalling axis maintains human breast luminal progenitor and breast cancer stem cells
Source: Oncogene. 2019 Jan 8;38(17):3151–69. doi: 10.1038/s41388-018-0656-7 (PMC6756022; doi:10.1038/s41388-018-0656-7)

**A**

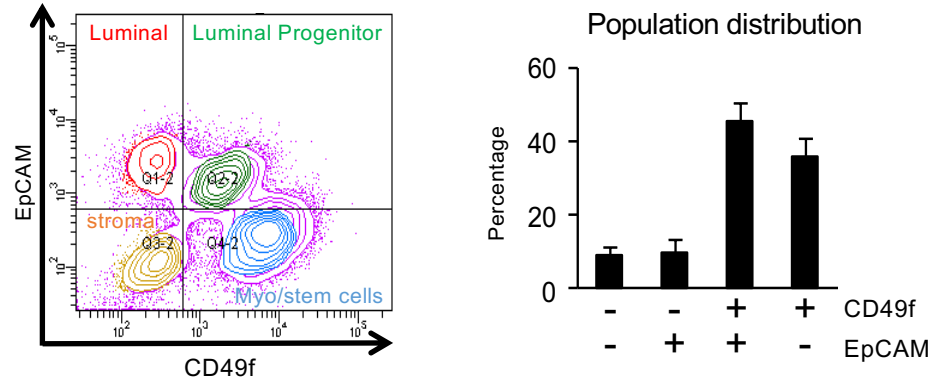

**B**

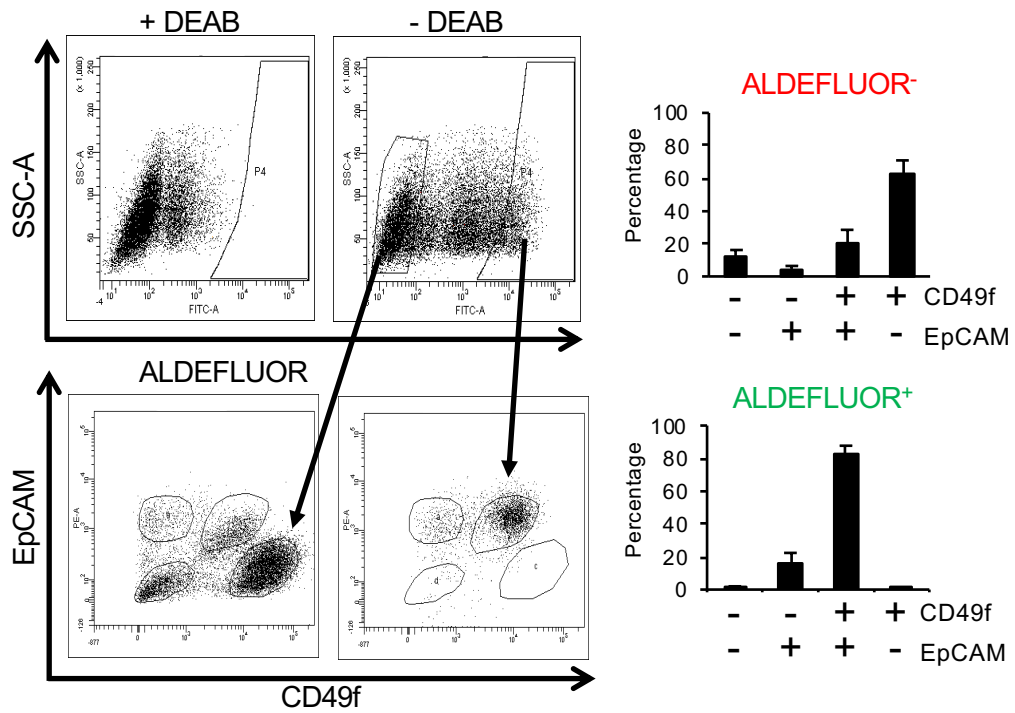

**C**

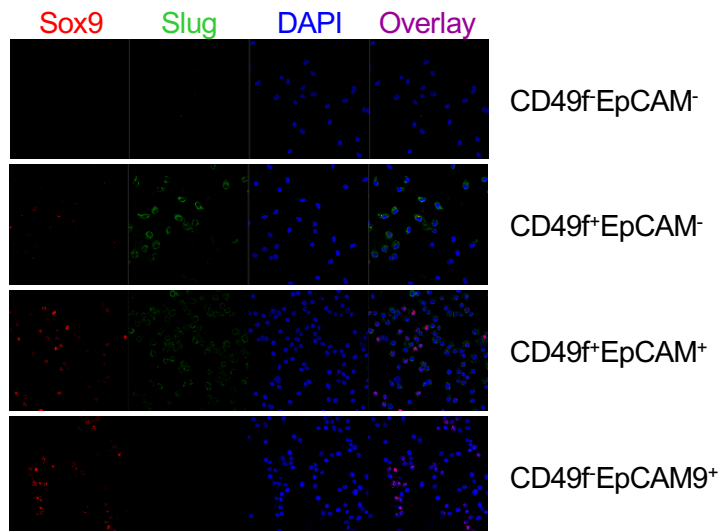

Supplement: Supplementary file 2 — Sup Fig 1 [file 41388_2018_656_MOESM2_ESM.pdf]

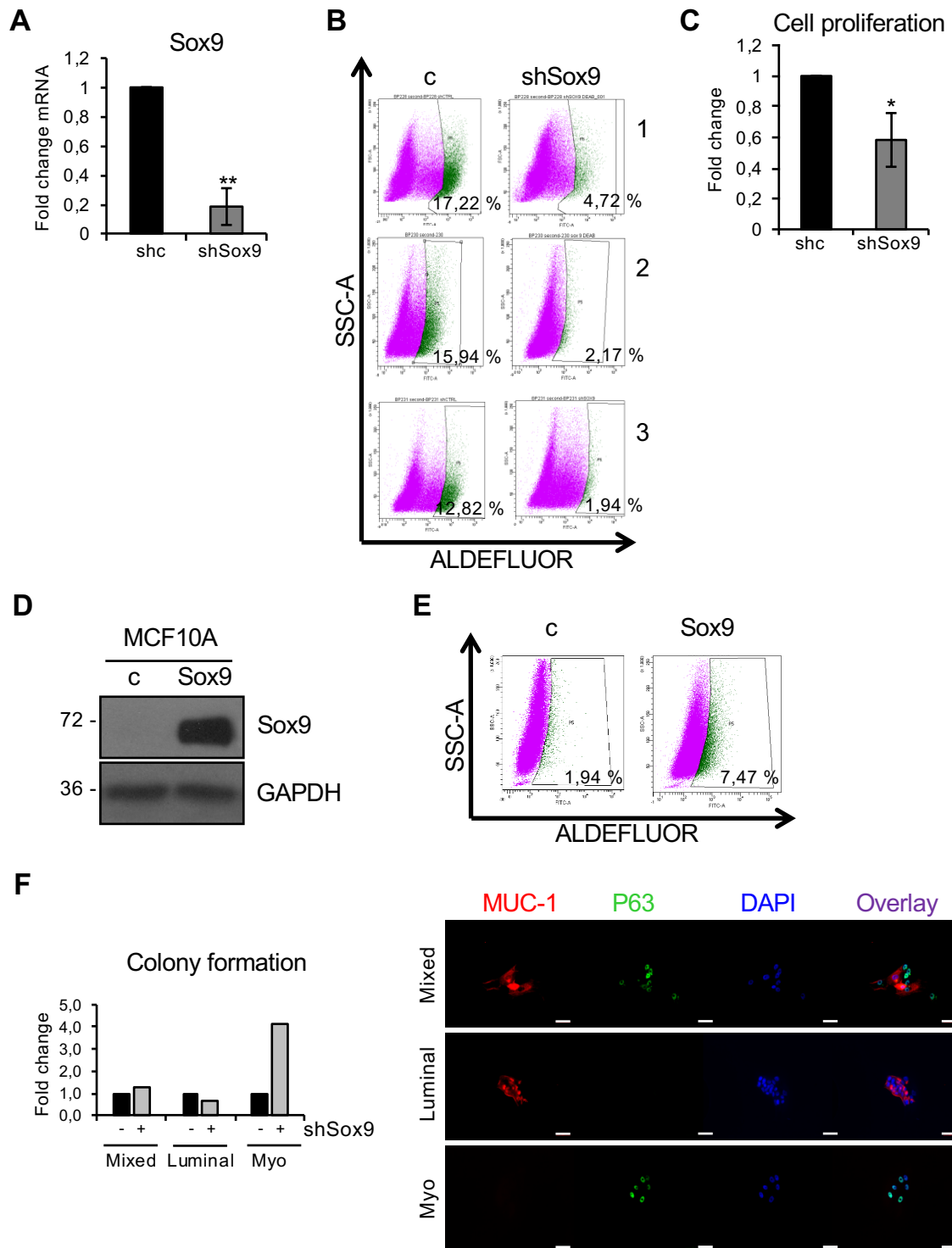

Supplement: Supplementary file 3 — Sup Fig 2 [file 41388_2018_656_MOESM3_ESM.pdf]

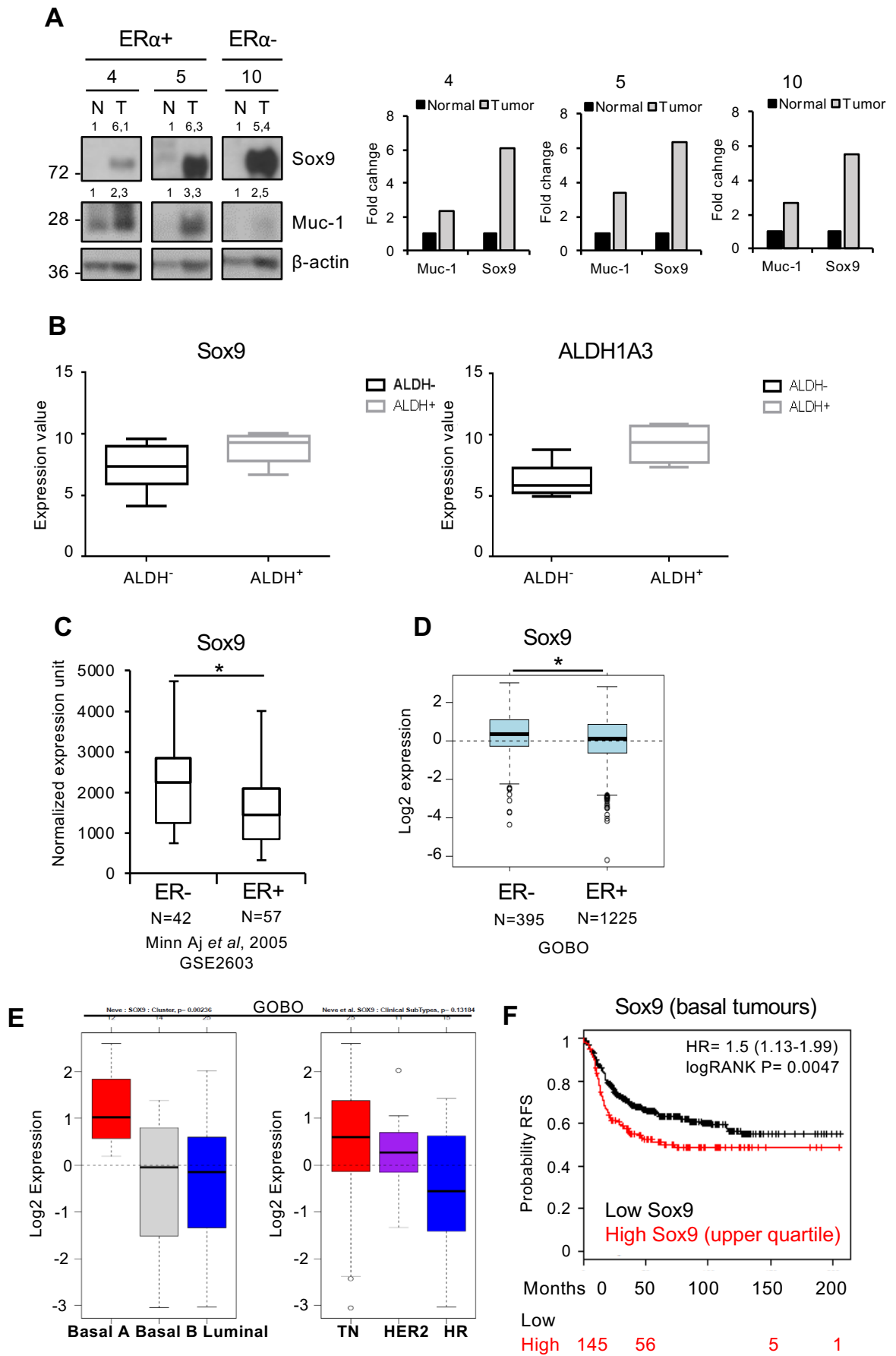

Supplement: Supplementary file 4 — Sup Fig 3 [file 41388_2018_656_MOESM4_ESM.pdf]

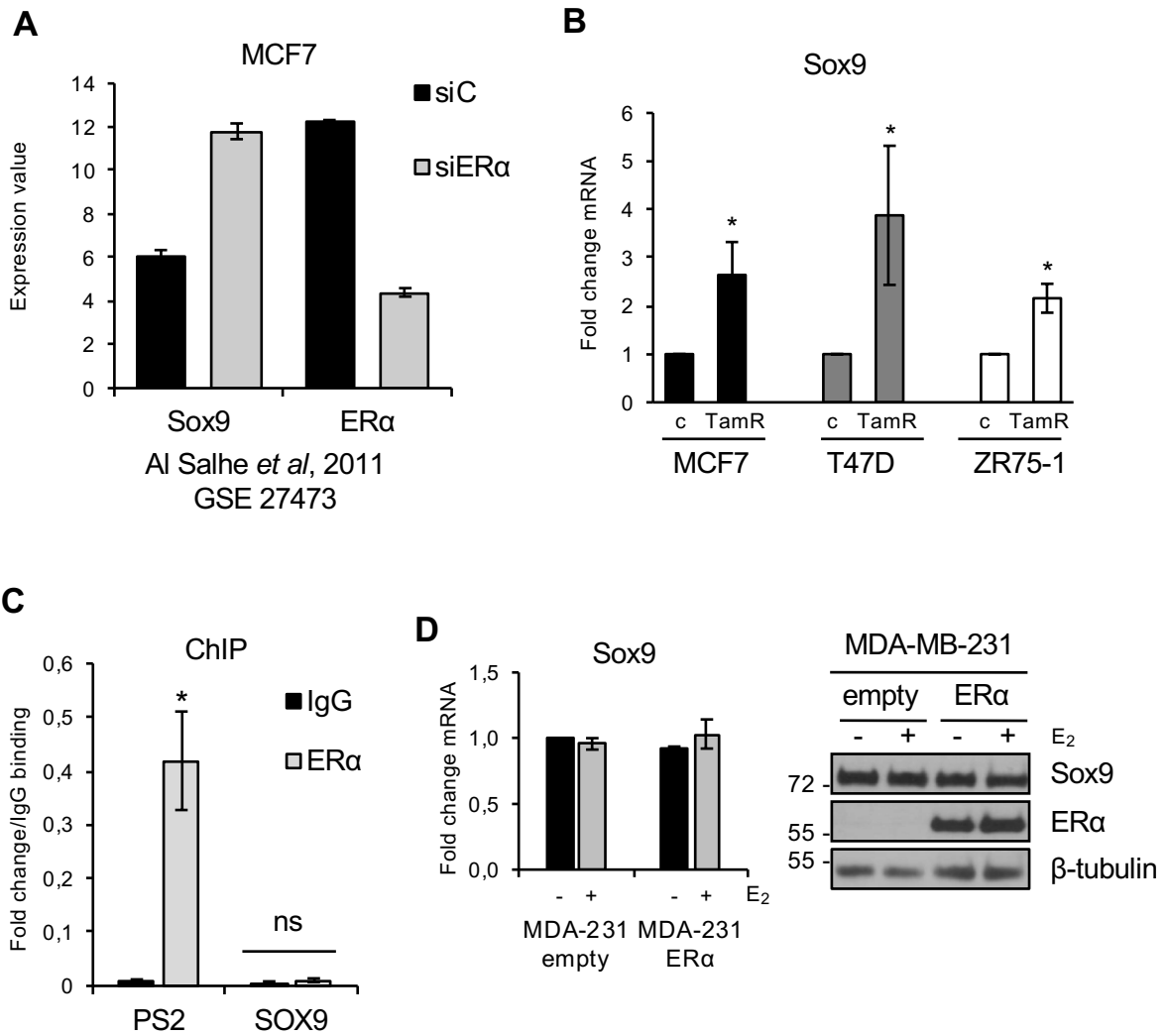

Supplement: Supplementary file 5 — Sup Fig 4 [file 41388_2018_656_MOESM5_ESM.pdf]

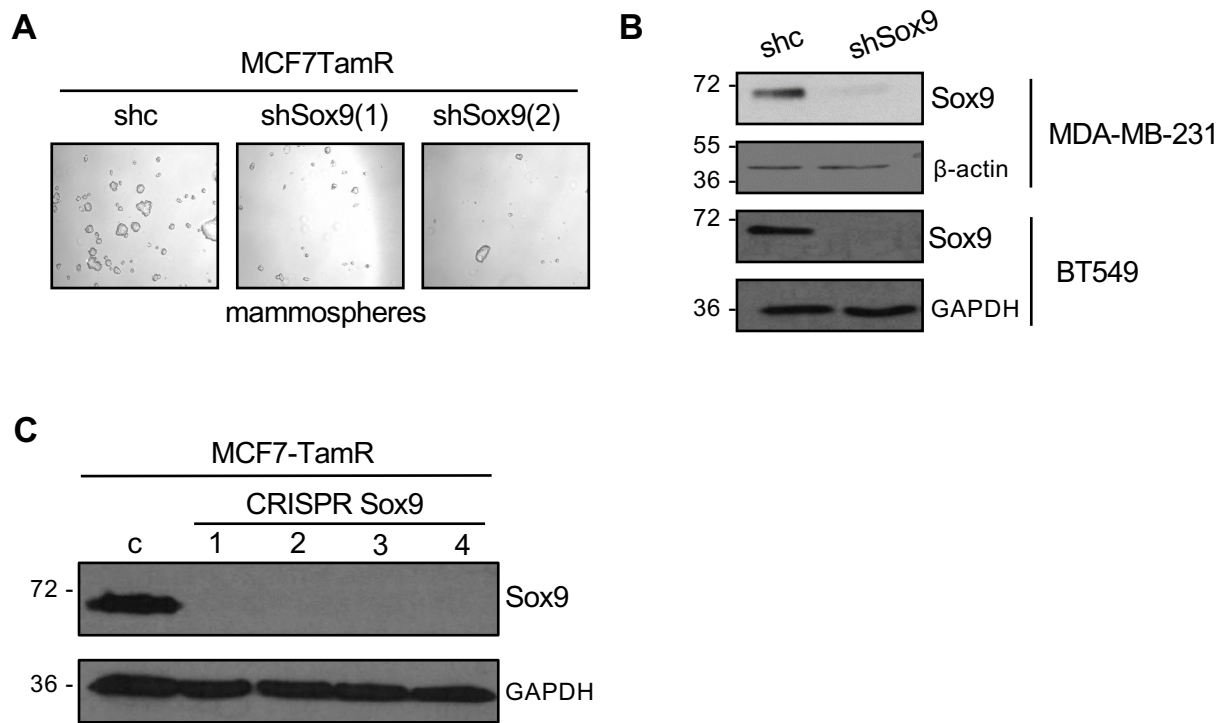

Supplement: Supplementary file 6 — Sup Fig 5 [file 41388_2018_656_MOESM6_ESM.pdf]

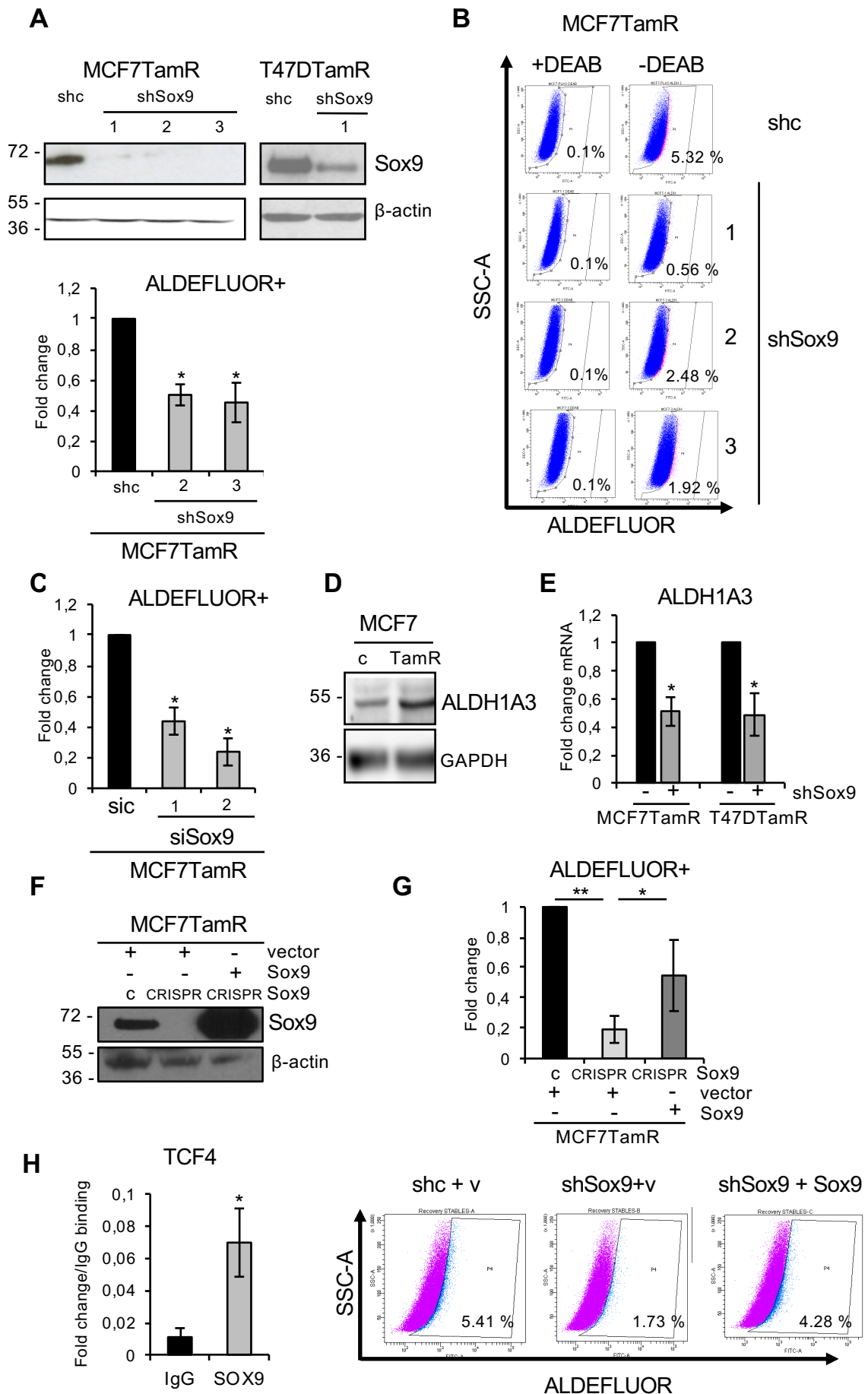

Supplement: Supplementary file 7 — Sup Fig 6 [file 41388_2018_656_MOESM7_ESM.pdf]

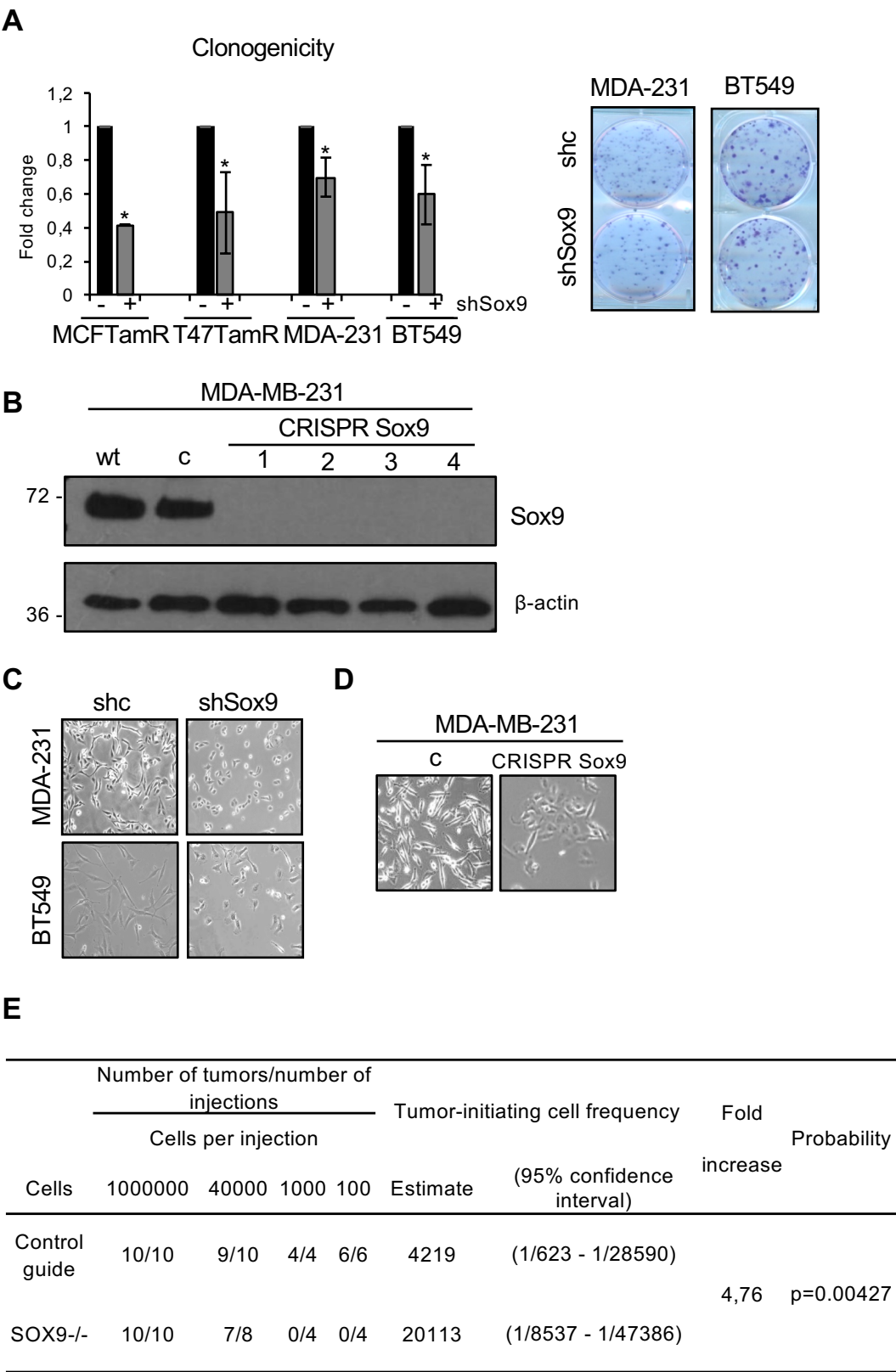

Supplement: Supplementary file 8 — Sup fig 7 [file 41388_2018_656_MOESM8_ESM.pdf]

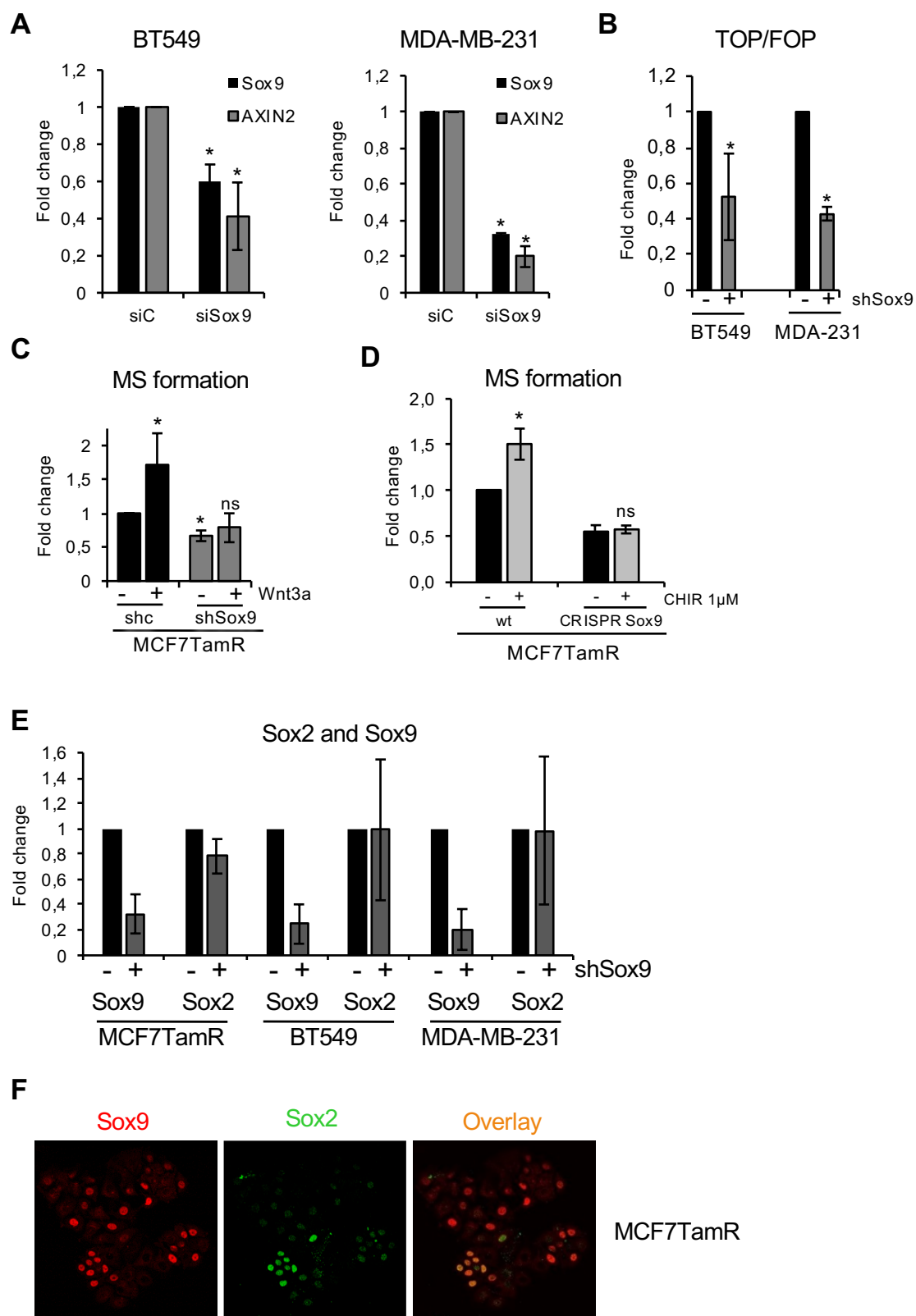

Supplement: Supplementary file 9 — Sup Fig 8 [file 41388_2018_656_MOESM9_ESM.pdf]
